# Comprehensive profiling of folates across polyglutamylation and one-carbon states

**DOI:** 10.1007/s11306-025-02269-5

**Published:** 2025-05-27

**Authors:** Sevcan Erşan, Yu Chen, Junyoung O. Park

**Affiliations:** 1 Department of Chemical and Biomolecular Engineering, University of California, Los Angeles, Los Angeles, CA 90095 USA; 2 Department of Chemistry and Biochemistry, University of California, Los Angeles, Los Angeles, CA 90095 USA

**Keywords:** Folate, One-carbon metabolism, Polyglutamylation, LC-MS, Metabolomics

## Abstract

**Introduction:**

One-carbon metabolism is central to carbon fixation, methylation, and biosynthesis of amino acids, lipids, and nucleotides. Folates are organic cofactors that harbor one-carbon units and shunt them across these metabolic pathways. Despite its essentiality to all life forms, the diverse nature of folate species with various polyglutamylation and one-carbon states makes their measurement challenging.

**Objectives:**

We aim to illuminate one-carbon metabolism by streamlining comprehensive profiling of folate polyglutamates.

**Methods:**

We analyze folate standards and cellular extracts containing diverse folates species by liquid chromatography-mass spectrometry (LC-MS).

**Results:**

We observe that *Escherichia coli* cells possess diverse folate polyglutamates with one to ten terminal glutamates. Interestingly, most folate polyglutamates form doubly charged ions as well as singly charged ions in LC-MS. Folates also undergo in-source fragmentation. The disparate fates of folates in MS make their quantitation prone to underestimation. Fragmentation by in-source collision-induced dissociation (CID) and LC separation circumvent this issue and facilitate robust and sensitive quantification of folates. In-source CID of folates generates reporter fragment ions that yield higher signals in the mass-to-charge ratio (m/z) range near the maximal mass resolution of Orbitrap MS. Our LC methods complement MS by effectively separating folates based on their polyglutamylation and one-carbon states.

**Conclusion:**

Our metabolomics approach tailored to folate polyglutamates reveals multiple layers of one-carbon metabolism organized by the lengths of polyglutamate tails in folates. Our analytical workflow is broadly applicable to folate profiling across various cell types to advance our knowledge of one-carbon metabolism as well as biotechnology and medicine.

**Supplementary Information:**

The online version contains supplementary material available at 10.1007/s11306-025-02269-5.

## Introduction

**Fig. 1:**
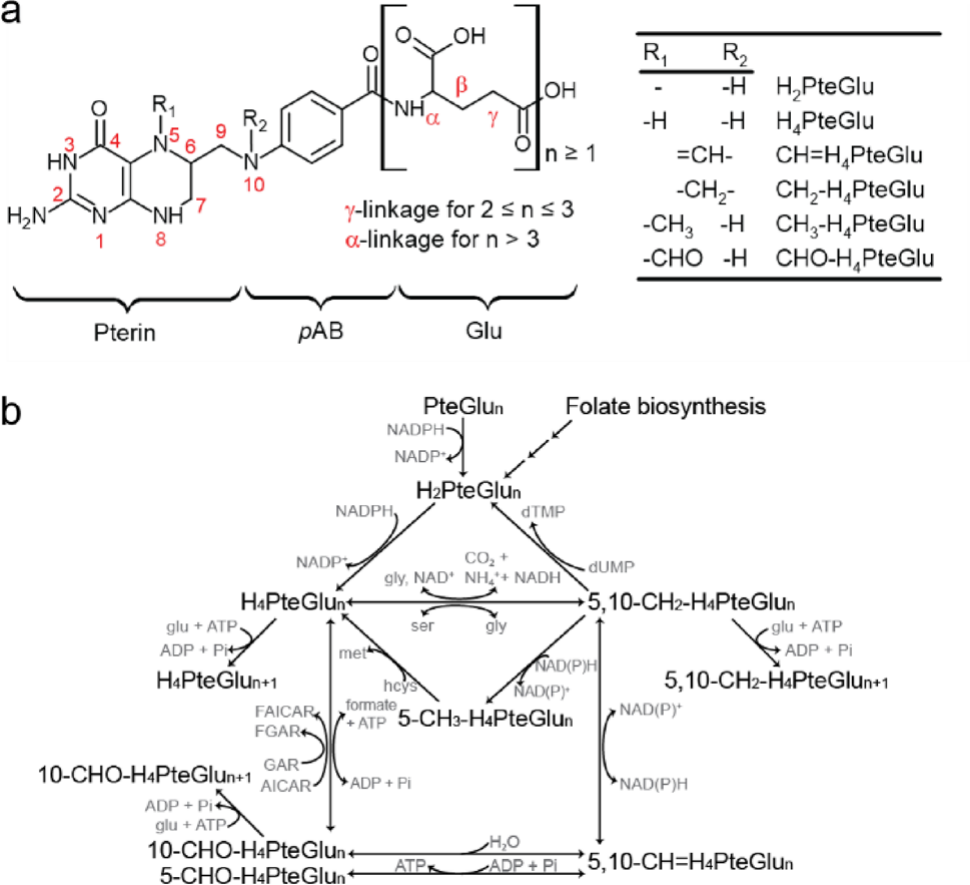
Structural diversity of folate polyglutamates in one-carbon metabolism. **a** Folates (PteGlu) are tripartite molecules composed of a pterin ring and a *p*-amino-benzoyl group (*p*AB), which are linked by a methylene bridge to form a pteroyl group (Pte), and glutamate (Glu). Pterin ring can be oxidized at R_1_ and N8 (PteGlu), reduced at R_1_ (H_2_PteGlu), and reduced at both R_1_ and N8 (H_4_PteGlu). The one-carbon units of various oxidation states (CH, CH_2_, CH_3_ or CHO) can attach at N5 or N10 positions (5-CH_3_-H_4_PteGlu, 5-CHO-H_4_PteGlu, and 10-CHO-H_4_PteGlu) or bridged in between (5,10-CH = H_4_PteGlu, and 5,10-CH_2_-H_4_PteGlu). Each folate species carries one or more terminal glutamates that constitute a polyglutamate tail. **b** In one-carbon metabolism, folates carry one-carbon units through redox reactions and interfaces with amino acid metabolism, nucleotide metabolism, and lipid metabolism. Folylpolyglutamate synthase catalyzes the ATP-dependent addition of glutamate to H_4_PteGlu_n_, 5/10-CHO-H_4_PteGlu_n_, and 5,10-CH_2_-H_4_PteGlu_n_

Folates encompass a broad spectrum of one-carbon carriers involved in amino acid and lipid metabolism as well as nucleotide synthesis and DNA methylation [1]. In acetogens, folates facilitate serial reduction of CO_2_ into a methyl group through the Wood-Ljungdahl pathway, also known as the reductive acetyl-coenzyme A pathway [2]. Each folate molecule consists of pterin, p-aminobenzoate, (poly)glutamate, and optionally a one-carbon unit (Fig. 1a) [3]. Folates are classified by the oxidation states of the pterin and the one-carbon unit as well as the degree of polyglutamylation.

Most folates inside the cell exist as polyglutamates containing as many as 14 glutamates and participate as coenzymes in one-carbon metabolism (Fig. 1b) [4, 5]. However, past research focused on folate monoglutamates due to their relevance to nutrition [6–9] and their presence in circulation [10]. An ability to reliably measure intracellular folate polyglutamates would advance our understanding of regulation of one-carbon metabolism in health and disease and promote metabolic engineering strategies for one-carbon utilization in biotechnology.

Various liquid chromatography-mass spectrometry (LC-MS) methods have been employed for measurement of folates (Table S1) [11–18]. However, comprehensive analysis of folate polyglutamates remains challenging due to a lack of their reference mass spectra, low intracellular concentrations [5, 13, 17], and instability [19, 20]. Folate polyglutamates can form diverse ion patterns in MS akin to peptides [21]. Adding to the challenge, ionization process in LC-MS often generates in-source fragments and adducts to obfuscate analyte’s identity and quantity [22].

We present LC-MS methods and a predictive LC retention model [23] for comprehensive profiling of folate polyglutamates. We used commercially available folate polyglutamates standards [24] to probe the effect of the length of polyglutamate tails on their retention in hydrophilic interaction chromatography (HILIC) and C18 reversed-phase chromatography. We generated their fragments using in-source collision-induced dissociation (CID) to yield mass spectra carrying structural information and common moieties useful for quantifying diverse folate species [25–29]. We demonstrate comprehensive folate quantification in *Escherichia coli*. Our analytical approach can be applied to other microorganisms as well as plant and mammalian systems to unveil the diversity and the unknown roles of folate polyglutamates.

## Materials and methods

### Folate standards and solution preparation

Authenticated standards of select folate species were obtained from Schircks Laboratories (Switzerland) (Table S2). *p*-Aminobenzoyl-glutamate (*p*ABGlu) and folic acid (PteGlu) were obtained from Sigma-Aldrich (St. Louis, MO). The standards represented all relevant species in one-carbon metabolism but 10-CHO-H_4_PteGlu_n_ due to their unavailability (Fig. 1). The standards were stored in a vacuum desiccator in a freezer until analysis. Stock standard solutions were prepared at 1 mM concentration in a solution of 2.5 mM sodium ascorbate, and 25 mM ammonium acetate at pH 7 [18]. Due to solubility limitations, Tetrahydrofolate (THF or H_4_PteGlu) was prepared at a concentration of 0.3 mM. Dihydrofolate (DHF or H_2_PteGlu) was dissolved in 1 mM ammonium acetate and 1 mM ammonium hydroxide at pH 9.4 following vendor recommendation. Brief ultrasonication was applied when necessary to ensure complete solubilization. For direct MS injection, standard solutions were prepared using the same solvent system but without sodium ascorbate.

### Microbial culture preparation and folate extraction

*Escherichia coli* strain K-12 strain MG1655 was cultured in Gutnick minimal medium [30] at 37 °C in a shaker incubator. A 4-mL sample of overnight culture was vacuum-filtered on a nylon membrane (25 mm diameter, 0.2 μm pore size, MilliporeSigma, Burlington, MA). The membrane was immediately transferred to a six-well plate containing 1 mL of precooled (4 °C) extraction solution (80:20 HPLC-grade acetonitrile/water with 2.5 mM sodium ascorbate and 25 mM ammonium acetate at pH 7) to quench metabolism and extract metabolites. Extraction continued for 20 min at 4 °C. The extract was transferred to a microcentrifuge tube and centrifuged at 17,000 x g for 10 min at 4 °C. The supernatant was transferred to a new tube, heated to 60 °C for 5 min to inactivate residual enzymes, and cooled on ice immediately after [18, 31]. The sample was dried under nitrogen gas flow, reconstituted in 40 µL of HPLC-grade water containing 25 mM sodium ascorbate and 25 mM ammonium acetate at pH 7, and transferred to an HPLC vial for LC-MS analysis.

### Direct infusion mass spectrometry

A hybrid quadrupole-Orbitrap mass spectrometer (Q Exactive Plus, Thermo Fisher Scientific) with heated electrospray ionization (HESI-II) probe was used for direct MS analysis of folate standards. MS was operated under the following conditions: spray voltage of 3.5 kV, a spray current of 1 µA, a capillary temperature set at 320 °C, the sheath gas with a flow rate of 28 au (arbitrary units), a 350 °C auxiliary gas with a flow rate of 10 au, S-lens RF level of 70, and a sweep gas flow rate of 0.98 au. Mass spectra were collected in both positive and negative ion modes over mass-to-charge ratios (m/z) between 100 and 1500, with a resolution of 140,000 at m/z 200, an automatic gain control (AGC) target at 3e6, and maximum injection time (IT) of 500 ms.

In-source CID activation energy ranging from 0 eV to 100 eV was applied in 10-eV increments. Higher energy collision dissociation (HCD) for MS2 was conducted using all ion fragmentation (AIF) with normalized collisional energy (NCE) from 10 to 50 in 10-unit increments. MS2 spectra were recorded at a resolution of 35,000 at m/z 200 following a full MS1 scan at a resolution of 70,000 at m/z 200.

Stock standard solutions of folate polyglutamates (prepared without sodium ascorbate) were diluted to 400 µM or 100 µM in 95:5 water/acetonitrile buffer at pH 9.4 (20 mM ammonium acetate and 20 mM ammonium hydroxide) or pH 4.0 (10 mM ammonium acetate with 0.1% acetic acid). These solutions were directly injected into the ESI source of MS at a flow rate of 50 µl/min.

Data processing involved analyzing the full scan MS1 and AIF MS2 spectra using Metabolomic Analysis and Visualization Engine (MAVEN, version 26.4) [32] and Xcalibur (version 4.2.47; Thermo Fisher Scientific), respectively. The top 10 most abundant peaks in MS1 spectra were selected after the removal of background noise and isotopic peaks. Additional expected (de)protonated and adduct ions were taken for further analysis.

### Liquid chromatography-mass spectrometry

LC-MS analysis was performed using a Vanquish Duo UHPLC system (Thermo Fisher Scientific) coupled to Q Exactive Plus MS. For LC, hydrophilic interaction liquid chromatography (HILIC) or a reversed-phase liquid chromatography (RPLC) were employed. Full MS scan over m/z 100–1500 in both positive and negative ion modes followed with a resolution of 140,000 at m/z 200, AGC target at 3e6, and max IT of 500 ms.

HILIC was adopted from an untargeted metabolomics method [33]. RPLC was adopted from Schober et al. [34]. For HILIC, an Xbridge BEH Amide XP column (130Å, 2.5 μm, 2.1 mm × 150 mm; Waters Corporation, Milford, MA) was used. For RPLC, a superficially porous amide-embedded C18 column (InfinityLab Poroshell 120 Bonus, 120Å, 2.7 μm, 2.1 mm × 150 mm; Agilent Technologies) was used. Both columns were maintained at 25 °C.

Two types of eluent A were used for HILIC. Basic eluent A (pH 9.4) consisted of 20 mM ammonium acetate and 20 mM ammonium hydroxide in 95:5 water/acetonitrile. Acidic eluent A (pH 4.0) consisted of 10 mM ammonium acetate with 0.1% acetic acid in 95:5 water/acetonitrile. Eluent B was acetonitrile. For RPLC, only the acidic eluent A (pH 4.0) was used, and eluent B was acetonitrile. Two segmented gradients were used for each LC type.

*HILIC method I. acidic/basic*: Both acidic (pH 4.0) and basic (pH 9.4) eluents were used. Chromatographic conditions were as follows: 90% B at 0 min, 90% B at 2 min, 75% B at 3 min, 75% B at 7 min, 70% B at 8 min, 70% B at 9 min, 50% B at 10 min, 50% B at 12 min, 25% B at 13 min, 25% B at 14 min, 0% B at 16 min, 0% B at 20 min, 90% B at 21 min, 90% B at 25 min. The total run time was 25 min with a flow rate of 150 µL/min. The sample injection volume of 5 µL.

*HILIC method II. acidic/basic*: Both acidic (pH 4.0) and basic (pH 9.4) eluents were used. Chromatographic conditions, modified after “HILIC method I” gradient, were as follows: 90% B at 0 min, 90% B at 2 min, 40% B at 22 min, 0% B at 25 min, 0% B at 28 min, 90% B at 30 min, 90% B at 40 min. The total run time was 40 min with a flow rate of 150 µL/min. The sample injection volume of 5 µL.

*RPLC method I*: Only acidic eluent (pH 4.0) was tested. Chromatographic conditions were as follows: 2% B at 0 min, 98% B at 20 min, 98% B at 25 min, 2% B at 26, 2% B at 35 min. The total run time was 35 min with a flow rate of 200 µL/min. The sample injection volume was 20 µL.

*RPLC method II*: Only acidic eluent (pH 4.0) was tested. Chromatographic conditions were as follows: 2% B at 0 min, 2% B at 2 min, 29% B at 8 min, 95% B at 10 min, 95%B at 12 min, 2% B at 14 min, and 2% at 20. The total run time was 20 min with a flow rate of 200 µL/min. The sample injection volume was 20 µL.

Chromatographic resolution ($\:{R}_{s}$) as an indication of LC separation of two analyte peaks was calculated using the following equation:

$$\:{R}_{s}=1.18\times\:\frac{{t}_{1}-{t}_{2}}{{w}_{\text{0.5,1}}+{w}_{\text{0.5,2}}}$$

where $\:{t}_{1}$ and $\:{t}_{2}$ are retention times of respective peak maxima, and $\:{w}_{\text{0.5,1}}$ and $\:{w}_{\text{0.5,2}}$ are the individual peaks’ full widths at half maxima (FWHM) in minutes [35].

Identification and quantification of metabolites were performed by processing LC-MS data using MAVEN [32] and comparing retention time and m/z to those of authenticated standards (Table S2) and an in-house database (Table S3). Extracted ion chromatograms (EIC) corresponding to individual m/z values of interest were exported.

### Regression analysis for LC retention

The relationship between LC retention times and the degrees of folate polyglutamylation was determined by log-linear regression analysis using retention time measurements from 5-methyltetrahydrofolate (CH_3_-THF or CH_3_-H_4_PteGlu) standards with one to four terminal glutamates. Retention times of CH_3_-THF with five and six glutamates were predicted from experimental values of 5-formyltetrahydrofolate (5-CHO-THF or 5-CHO-H_4_PteGlu) standards with five and six terminal glutamates by assuming a constant retention time offset between 5-CHO-THF and CH_3_-THF across all polyglutamate forms. The model of LC retention times for folate polyglutamates was formulated as follows:

$$\:y=\:{\beta}_{0}+{\beta}_{1}\:In\:\left(x\right)+\varepsilon$$

where $\:y$ represents the retention time in minutes, $\:x$ is the number of glutamates in the folate species. ${\beta}_{0}$ and ${\beta}_{1}$ are constants representing the intercept and slope of the regression line, respectively. $\varepsilon$ represents a retention time shift specific to the monoglutamate form of each folate species of interest in reference to CH_3_-THF, for which $\varepsilon=0$.

## Results and discussion

### Adducts and doubly charged ions of folate species are prevalent in direct infusion MS

**Fig. 2:**
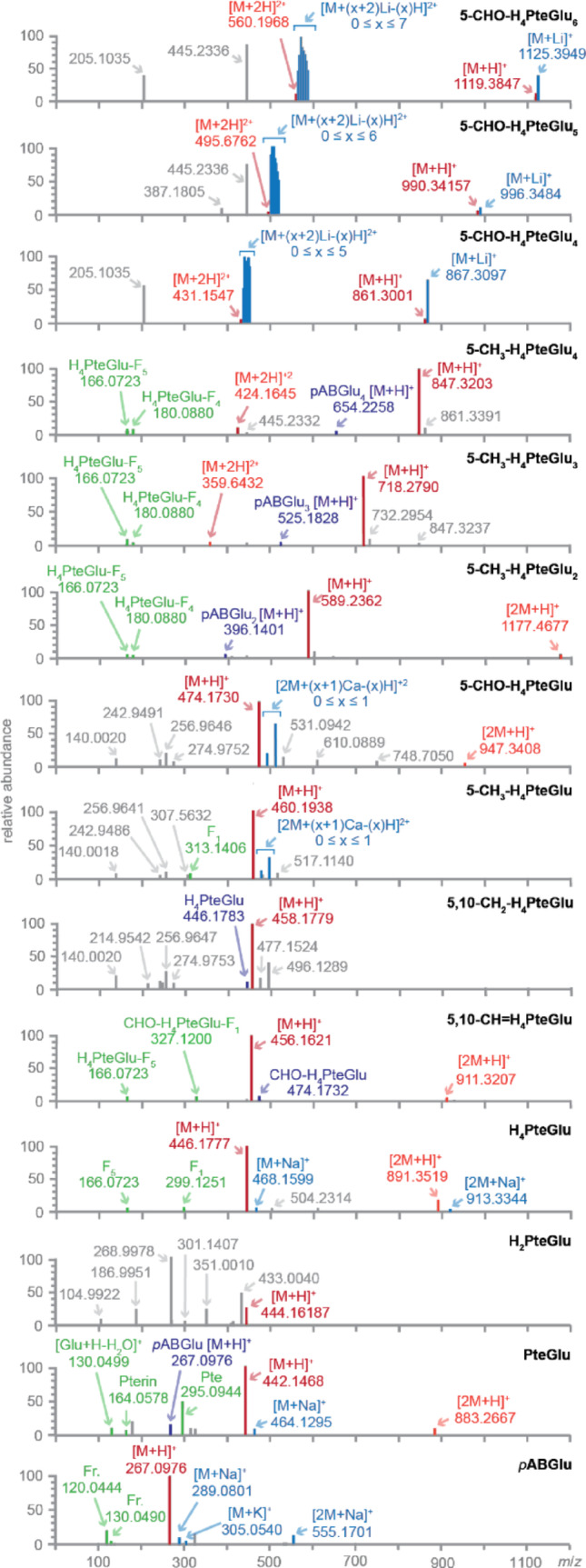
The top 10 most abundant ions in direct infusion mass spectrometry of folate polyglutamates. Folate polyglutamates with three or more terminal glutamates were doubly charged [M + 2 H]^2+^. Cationic adducts of sodium, potassium, calcium, and lithium were observed. Mass spectra were collected in full scan positive ion mode. Precursor ion peaks are denoted in red, cationic adducts in blue, interconversion and degradation products in purple, dimer ions in pink, fragment ions in green, and unidentified peaks in grey

We sought to identify ions that offer sensitive detection and accurate identification of folate polyglutamates. We characterized the mass spectra resulting from the direct injection of folate standards, which eliminated potential artifacts and interferences that may result from folate instability during liquid chromatography [19, 20]. Full scan MS data were collected to detect comprehensive ion features within a m/z range from 50 to 1500. For each standard injection, we examined the total ion current (TIC) chromatogram and focused on the top 10 peaks with the highest signal intensities (Fig. 2).

Electrospray ionization of folate polyglutamates generated diverse ion products, including singly and doubly charged monomeric and dimeric forms in both positive and negative mode (Fig. 2 and Figs. S1-3). Signal intensities were comparable between positive and negative mode (Table S4). Adduct formation with alkali metals was observed. The type and relative abundance of these adducts depended on folate species, length of polyglutamate tail, and matrix composition. Adduct formation was particularly prevalent in CHO-THF with four to six terminal glutamates with singly and doubly charged lithium adducts surpassing the abundance of [M + H]^+^, [M + 2 H]^2+^, [M-H]^−^, and [M-2 H]^2−^ ions (Fig. 2 and Figs. S1-3). While the high abundance of lithium adduct was due to folate polyglutamate standards being prepared as lithium salts, other cation adducts such as sodium, potassium, and calcium were also observed.

**Fig. 3:**
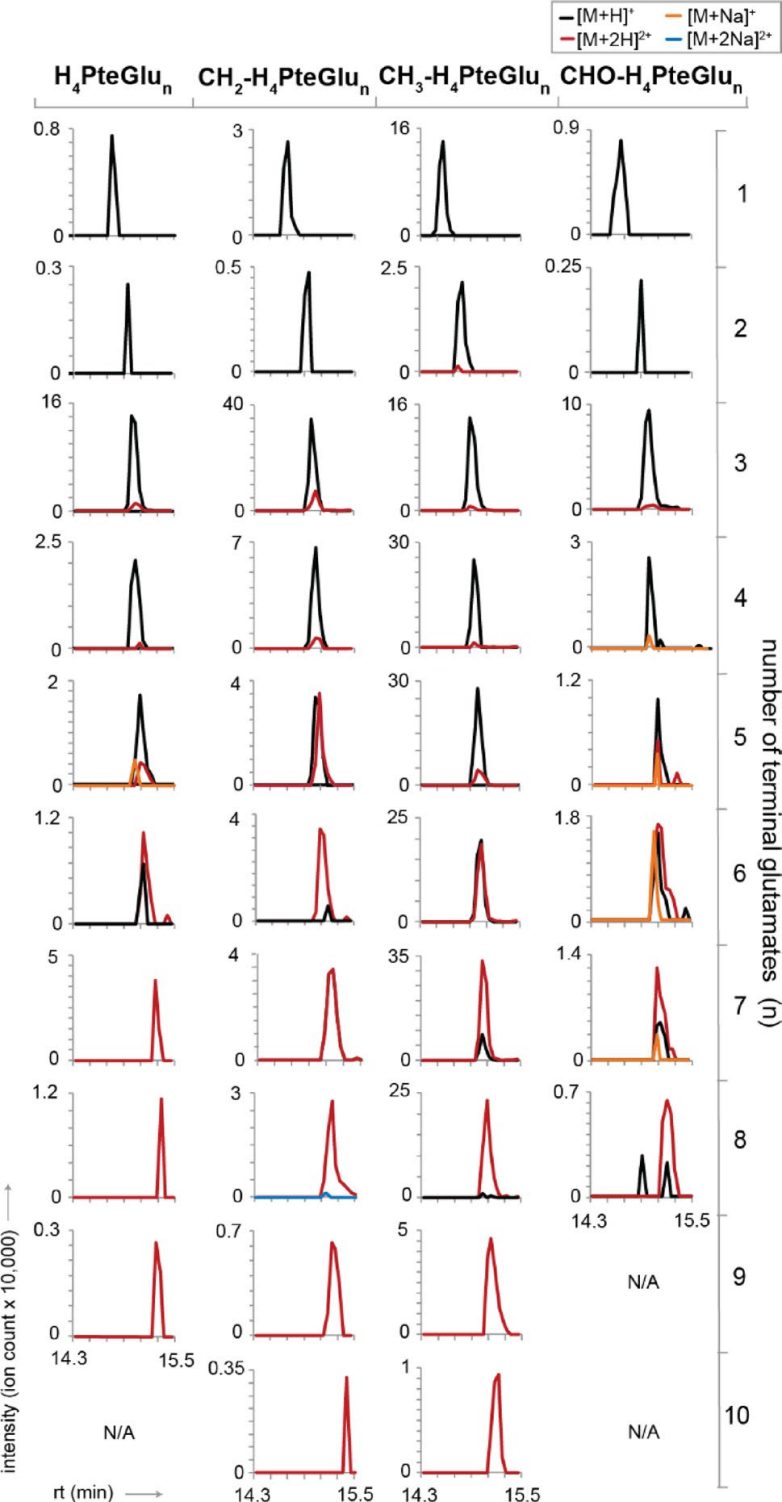
LC-MS analysis of folate polyglutamates in *E. coli*. Metabolite extract from *E. coli* was analyzed on LC-MS set up with HILIC method I, basic eluent, and positive ion mode. CHO-H_4_PteGlu_n_ represented isomers 5-CHO-H_4_PteGlu_n_ and 10-CHO-H_4_PteGlu_n_. Doubly charged ions [M + 2 H]^2+^ became more abundant as the number of terminal glutamates (n) in folate polyglutamates increased. The abundance of doubly charged ions overtook that of singly charged ions with *n* ≥ 6. The plots show LC-MS analysis of folate species from one *E. coli* culture. The folate profiles including doubly charged ions were reproducible in a biological replicate

Folate polyglutamates with three or more terminal glutamates displayed doubly charged parent ions [M + 2 H]^2+^ (Fig. 2), consistent with a previous observation [18]. Folate polyglutamates even with long polyglutamate tails were only singly or doubly charged unlike peptides which may have higher charge states under ESI [36]. The abundance of doubly charged ions became more prominent with longer polyglutamate tails, surpassing those of singly charged ions upon reaching six terminal glutamates.

### Cell lysates abound in doubly charged ions and sodium adducts of folate polyglutamates

We profiled folates in *E. coli* by LC-MS. In positive ion mode, we detected 38 tetrahydrofolate species with one to 10 terminal glutamates (Fig. 3). Tetrahydrofolates with one to four terminal glutamates were mainly observed as singly charged ions [M + H]^+^. Folate polyglutamates with seven to 10 terminal glutamates were detected almost exclusively as doubly charged ions [M + 2 H]^2+^. Sodium adducts [M + Na]^+^ were prevalent for CHO-THF polyglutamates but not for other types of folates. Sodium adducts were abundant despite Gutnick minimal medium not containing sodium likely due to the quenching and extraction solution containing sodium ascorbate. The formyl group of CHO-THF in conjunction with the carboxyl-rich polyglutamate tail, which can readily interact with cations [37], may be responsible for stabilizing the sodium adduct. Thus, detection and quantification of folate polyglutamates in biological samples warrant examining doubly charged ions and monovalent and divalent cation adducts.

Based on ion counts, CH_3_-THF was the most abundant, especially with four to eight terminal glutamates. For other folate species THF, CHO-THF, and methylenetetrahydrofolate (CH_2_-THF or CH_2_-H_4_PteGlu), polyglutamates with three terminal glutamates were most abundant, consistent with prior observations [17, 18]. Folate species with more than 10 terminal glutamates were not detected in *E. coli* lysate. In erythrocytes and plants, folates with as many as 11 and 14 terminal glutamates, respectively, have been observed [4, 5]. These variations in the lengths of glutamate tails across different cell types could be attributed to biological differences such as cell type and physiological state [38, 39]. Improved folate extraction and instrument sensitivity may lead to robust quantification of even larger folate polyglutamates.

### Folate species undergo in-source fragmentation and interconversion

We detected several prominent peaks besides adducts and doubly charged ions in direct infusion mass spectrometry of folate standards (Fig. 2 and Figs. S1–3). Given that the standard solutions had been freshly prepared just before MS analysis, these peaks suggested in-source modifications. To distinguish between in-source fragmentation and in-sample degradation, we put the standards through LC-MS. If the fragment ions had the same retention times as the parent ions, we determined that the fragments were formed in the source. Otherwise, the “fragment” peaks would have had to come from in-sample (or on-column) degradation.

**Fig. 4:**
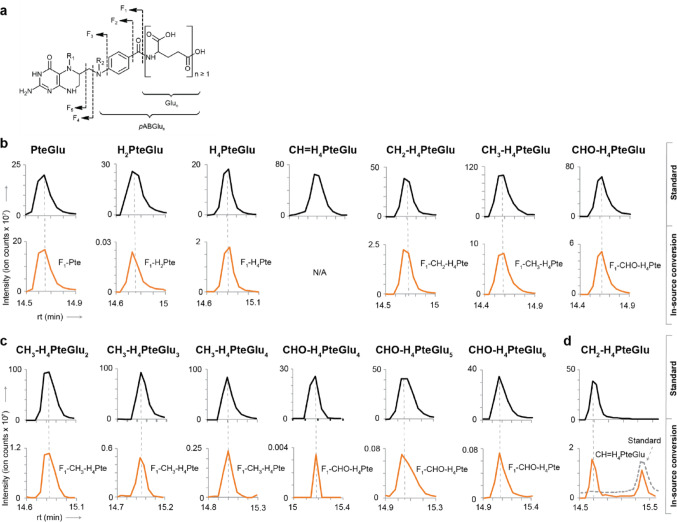
In-source fragmentation of folate polyglutamates in LC-MS. (**a**) LC-MS analysis of folate polyglutamates revealed various fragments including those shown as F_1_ through F_5_. (**b**) In-source fragmentation of various folate monoglutamate species produced F_1_ fragments, whose LC retention times were the same as those of the parent ions. (**c**) In-source fragmentation of various folate polyglutamates produced F_1_ fragments. (**d**) LC-MS of CH_2_-H_4_PteGlu standard also produced two CH = H_4_PteGlu peaks. The left peak shared the same retention time with CH_2_-H_4_PteGlu, suggesting its in-source oxidation. The retention time of the right peak aligned with that of CH = H_4_PteGlu standard (dashed gray line), indicating the in-sample or on-column conversion of CH_2_-H_4_PteGlu

LC-MS analysis of folate polyglutamate standards revealed their in-source fragments formed by neutral loss of glutamate residues. In positive ion mode, folate polyglutamates produced a common fragment ion F_1_ at m/z 313.1408 and m/z 327.1200 for CH_3_-THF and CHO-THF polyglutamates, respectively (Fig. 4a and Table S5). The consistent formation of F_1_ fragments across all folate standards suggested their potential use as reporter ions to identify and quantify folate species of varying one-carbon oxidation state (Fig. 4b) regardless of their polyglutamylation state (Fig. 4c). Targeting the F_1_ fragments would improve quantification accuracy and spectral accuracy by allowing a narrow scan range that targets only these fragment ions rather than the broad mass range of folate polyglutamates [40].

Interestingly, with the CH_2_-THF standard, we observed both in-source and in-sample conversion of its one-carbon unit to form methenyltetrahydrofolate (CH = THF or CH = H_4_PteGlu) (Fig. 4d). Of the two distinct peaks detected in CH = THF mass channel obtained upon injecting the CH_2_-THF standard to LC-MS, the earlier peak shared the same retention time as the CH_2_-THF standard, indicating in-source oxidation of CH-H_4_PteGlu. The latter peak corresponded to in-sample or on-column oxidation of the CH_2_-THF standard into CH = THF. Folates undergo interconversion and degradation in solution without proper precautions [19, 20, 41]. While chemical derivatization and elimination of residual enzyme activity mitigate these pitfalls [41], in-source and in-sample conversion may confound accurate identification and quantification of folates in biological samples (Supplementary Note 1).

### In-source CID and MS2 generate reporter ions for sensitive folate quantification

**Fig. 5:**
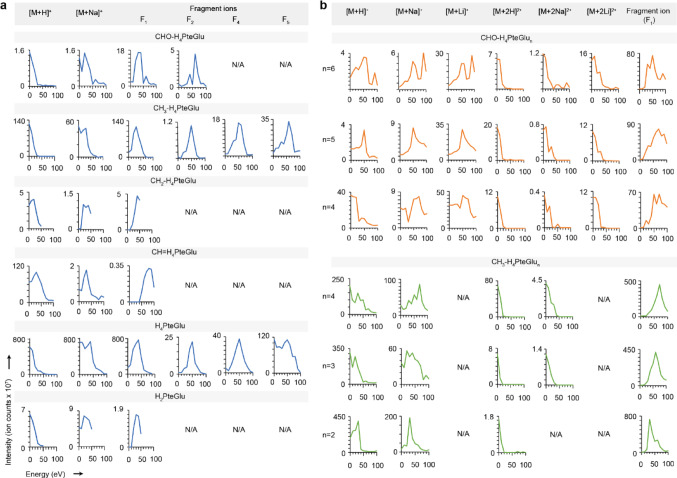
Fragmentation products from in-source CID of folate polyglutamates. **a** Upon direct infusion mass spectrometry and in-source CID of folate monoglutamates, the parent ions and cation adducts as well as F_1_, F_2_, F_4_, and F_5_ fragments were observed under ion kinetic energies ranging from 0 to 100 eV. **b** The same in-source CID of folate polyglutamates generated singly and doubly charged ions as well as F_1_ fragments

While the F_1_ in-source fragments showed promise for identifying and quantifying folates across polyglutamylation and one-carbon state, they were not as abundant as the parent ions [M + H]^+^, thus posing a shortcoming in the lower limit of detection (Fig. 4b, c). To improve F_1_ fragment formation, we tested in-source CID with direct infusion MS. With ion kinetic energies ranging from 30 eV to 50 eV, we observed strong peaks for the F_1_ fragment ions of all folate species except for CH = THF (Fig. 5a). For CHO-THF, CH_3_-THF, and THF, we observed F_2_, and for CH_3_-THF, and THF, we also observed F_4_, and F_5_ with their strongest signals coming at or above 50 eV. Glutamate was also observed as a fragmentation product for all THF species but CH_2_-THF (Fig. S4a). As ion kinetic energies increased, the signals for parent ions and sodium adducts decreased. For polyglutamated forms of CHO-THF and CH_3_-THF, the F_1_ fragment ions displayed the strongest signals across all tested lengths of polyglutamate tails (Figs. 5b and S4b).

Fragmentation by AIF on MS2 was performed for comparison (Figs. S5 and S6). On MS2, the F_1_ ions, resulting from the loss of Glu_n_ from folates, were consistently among the most abundant across all folate polyglutamates. Unique to CH_2_-THF, fragment ion F_3_ was observed. For CH = THF, we did not observe much discernible fragmentation across the tested NCE range. These observations suggested that the one-carbon bridges between N5 and N10 in CH_2_-THF and CH = THF lead to fragmentation patterns different from other folate species. The strong signals for F_1_ fragment ions in both in-source CID and AIF MS2 indicated their utility as sensitive quantification and accurate identification of various folate species regardless of polyglutamate tail lengths. An added benefit of F_1_ fragment ions was their small sizes amenable to near-maximal mass resolution in Orbitrap-based MS.

### HILIC separates folate polyglutamates with different numbers of terminal glutamates

The F_1_ fragments are useful reporter ions because their structures are distinct for DHF, THF, and those THF molecules that harbor different one-carbon units. Folate species that are different only in the lengths of their polyglutamate tails may be distinguished by the parent ions in MS1 or in MS/MS but not by in-source CID because their F_1_ fragments are identical. Thus, we needed to separate folate polyglutamates by LC.

**Fig. 6:**
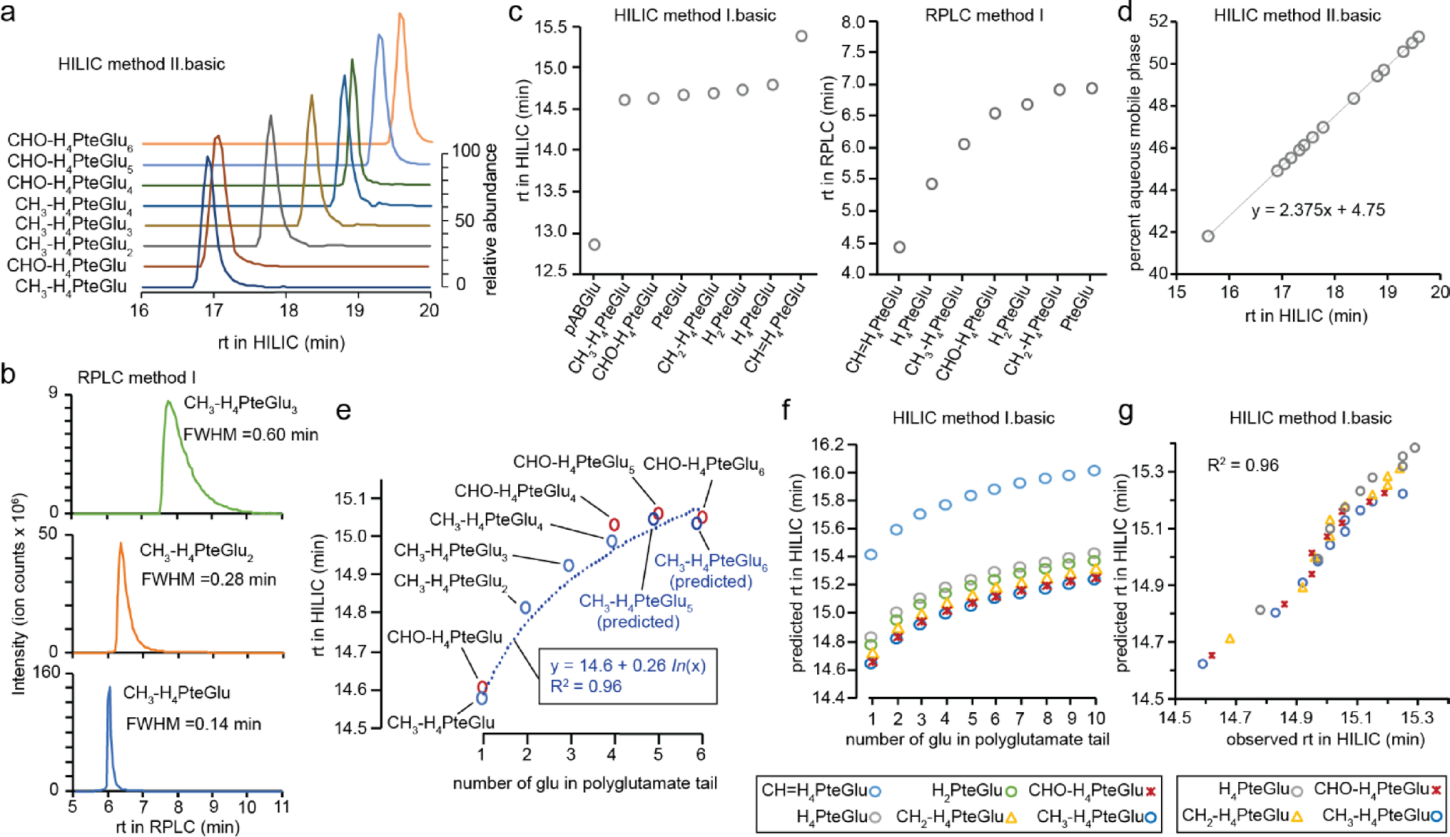
LC retention behavior of folate polyglutamates. **a** HILIC method II effectively separated folate polyglutamates based on the numbers of their terminal glutamates. **b** While RPLC method separated folate polyglutamates, peak broadening occurred as the number of terminal glutamates increased. **c** Both HILIC and RPLC methods separate folate monoglutamates. Although folate monoglutamates eluted earlier in RPLC, the retention time differences between them were larger. **d** All tested folate standards eluted between 40% and 52% aqueous mobile phase in HILIC. A higher percent of aqueous mobile phase is expected to elute folates with longer polyglutamate tails. **e** The retention times of folate polyglutamates followed a log-linear relationship with respect to the number of terminal glutamates. **f** Taking into consideration the baseline differences in folate monoglutamates, the retention times of folates species with various one-carbon units and polyglutamylation in HILIC were predicted. **g** For the polyglutamate forms of four folate species THF, CHO-THF, CH_2_-THF, and CH_3_-THF, the observed retention times matched with the predicted retention times

We measured the retention times of folate standards on HILIC and RPLC followed by MS (Fig. S7a, b and Table S6). For HILIC eluent A buffers were at pH 4.0 and 9.4, and for RPLC, eluent A buffer was at pH 4.0. We tested different solvent gradients to separate in-source modification from in-sample degradation (Supplementary Note 2). Overall, HILIC retained and separated folate species better than RPLC (Fig. 6a and Table S7). Peak widths in RPLC, indicated by the FWHM increased from 0.14 min to 0.60 min as the number of terminal glutamates increased from one to three (Fig. 6b). In both HILIC and RPLC, increasing lengths of polyglutamate tails increased the retention times of folates.

In addition to polyglutamylation, the pterin ring and the one-carbon unit of folates affected LC retention. Reduction of pterin ring, from folate (PteGlu) to DHF (H_2_PteGlu) to THF (H_4_PteGlu), led to longer retention in HILIC but shorter retention in RPLC (Fig. 6c). In HILIC, the tested folate standards eluted between 40% and 52% aqueous mobile phase in the solvent gradient (Fig. 6d). Since more terminal glutamates increased the retention of folate species, > 52% aqueous mobile phase may be required to elute longer polyglutamates. Thus, combined LC-MS (including in-source CID) would lead to robust and accurate measurement of folate polyglutamates.

### Folate polyglutamate LC retention prediction and validation in vivo

Since not all folate polyglutamate standards are readily available, we developed an LC retention model to predict the retention times of any folate polyglutamates. To this end, we performed regression analysis using available CH_3_-THF and CHO-THF polyglutamate standards (Fig. 6e). We observed a log-linear relationship between the number of terminal glutamates and the retention time of folate polyglutamates in HILIC with a high coefficient of determination (R^2^ = 0.96). To account for the baseline retention time differences between folate monoglutamates, we added a retention time shift (ε) of each folate species relative to CH_3_-THF, for which ε = 0. The revised model was then employed to predict the LC retentions of six folate species with as many as 10 terminal glutamates (Fig. 6f). For polyglutamated forms of THF, CHO-THF, CH_2_-THF, and CH_3_-THF, the predicted retention times in HILIC were consistent with measurements from *E. coli* lysate (R^2^ = 0.96) (Figs. 6g and S8a). On the other hand, we observed discrepancies between observed and expected retention times of CH = H_4_PteGlu_n_ and H_2_PteGlu_n_ (Fig. S8b). The inaccurate prediction for the CH = THF and DHF species was due to the subdued effect of additional terminal glutamates on folate retention that results in insufficient separation. In the *E. coli* lysate samples, all detected DHF polyglutamates but one were observed within 0.4 min of each other, and all detected CH = THF polyglutamates were observed within 0.1 min of each other.

## Conclusion

In the present study, we comprehensively characterized the chromatographic behavior, ionization, and in-source conversion of folates with varying polyglutamylation and one-carbon states in LC-MS. The presented LC-MS strategies enabled sensitive and robust quantification of folate polyglutamates across ranges of one-carbon units and terminal glutamates. As most cellular folates exist in polyglutamate forms, we expect our methods to advance our knowledge of one-carbon metabolism. Specifically, we can now start thinking about folate species with the same polyglutamate tail length occupying a layer of one-carbon metabolism and probe if carbon fluxes differ across layers, which may serve different purposes. For example, reductive one-carbon metabolism may utilize different layers (i.e., different pools of folate polyglutamates) than oxidative one-carbon metabolism as carbon flux may be more streamlined within folate pools that have the same or similar numbers of terminal glutamates. Thus, profiling cellular folate polyglutamates will help elucidate multilayer one-carbon metabolism in carbon-fixing organisms and in human disease models, advancing sustainable biotechnology and medicine.

## Electronic supplementary material

Below is the link to the electronic supplementary material.

Supplementary Material 1

## Data Availability

Data is provided within the manuscript or supplementary information files. Raw data files are available from the corresponding author on reasonable request.

## Abbreviations

LC-MS: Liquid chromatography-mass spectrometry
ESI: Electrospray ionization
pAB: p-aminobenzoate
Pte: Pteroyl group or pteroic acid
Glu: L-glutamate
pABGlu: p-aminobenzoyl-glutamate
PteGlu: Pteroylglutamate (folate)
PteGlu_n_: Pteroylpolyglutamate (folate polyglutamate)
H_2_PteGlu: Dihydropteroylglutamate (dihydrofolate)
DHF: Dihydrofolate
H_4_PteGlu: Tetrahydropteroylglutamate (tetrahydrofolate)
THF: Tetrahydrofolate
(5-/10-)CHO-H_4_PteGlu or (5-/10-)CHO-THF: Formyltetrahydrofolate
(5: 10-)CH = H_4_PteGlu or (5,10-)CH = THF: Methenyltetrahydrofolate
(5: 10-)CH_2_-H_4_PteGlu or (5,10-)CH_2_-THF: Methylenetetrahydrofolate
(5-)CH_3_-H_4_PteGlu or (5-)CH_3_-THF: Methyltetrahydrofolate
