# Supplementary material for: Comprehensive profiling of folates across polyglutamylation and one-carbon states
Source: Metabolomics. 2025 May 27;21(3):71. doi: 10.1007/s11306-025-02269-5 (PMC12116830; doi:10.1007/s11306-025-02269-5)
Supplement: Supplementary file 1 — Supplementary Material 1 [file 11306_2025_2269_MOESM1_ESM.docx]

Supplementary Information for

# **Comprehensive Quantitation of Folates Across Polyglutamylation and One-Carbon States**

Sevcan Erşan^1^, Yu Chen^2^, and Junyoung O. Park^1^*

^1^ Department of Chemical and Biomolecular Engineering, and

^2^ Department of Chemistry and Biochemistry, University of California, Los Angeles, Los Angeles, CA 90095, USA.

* Correspondence: [jop@ucla.edu](mailto:jop@ucla.edu)

**Table of Contents** page

Supplementary Note 1…………………………………………………………………………...2

Supplementary Note 2…………………………………………………………………………...3

Supplementary Figures S1-S8……………………………………………………………….....4

Supplementary Table S1-S7....………………………………………………………………..12

Supplementary References……………………………………………………………………26

**Supplementary Note 1. Interconversion and degradation products of folate polyglutamates in LC-MS**

The detection of interconversion folate products in direct injection mass spectrometry led us to investigate their origins with a focus to understand the extent and the significance of these transformations. Furthermore, we set out to probe how HILIC method that is commonly employed with basic mobile phase [1–3] affects analysis if acidic mobile phase is used in terms of stability, chromatographic separation, and MS detection as folates are reported to undergo pH dependent interconversion and degradation reactions.

To achieve this, individually prepared folate standards were injected into LC-MS. For each standard, we examined m/z values corresponding to all folate species as well as degradation products including the pteroyl group, pterin derivatives, and folate polyglutamates with fewer terminal glutamates than the parent ions. The data helped us determine if interconversion, degradation and fragmentation occurred in the sample or in the ion source. When other product ions exhibited the same chromatographic retention times to their parent ions, it indicated in-source conversion. If the retention times differed, it indicated in-sample or in-column conversion.

Folate species underwent an oxidation process on the pterin ring, yielding interconversion folate products. Formation of these products were found to be dependent on folate type and polyglutamylation. Specifically, H_2_PteGlu, H_4_PteGlu, and CH_2_-H_4_PteGlu exhibited a range of oxidized folate forms. PteGlu, CH_3_-H_4_PteGlu, and CHO-H_4_PteGlu_n_ standards were stable and did not produce substantial peaks corresponding to other ions. In contrast, CH_3_-H_4_PteGlu_n_ underwent oxidation to CH_2_-H_4_PteGlu_n_, indicating an increased susceptibility to oxidation with a longer polyglutamate tail. Folate polyglutamate standards (PteGlu_n_) displayed deglutamylation products PteGlu_n-1_. A common oxidative cleavage product *p*ABGlu_n_ was identified across all folate polyglutamate injections with their retention times consistent with the *p*ABGlu standard. This alignment suggests their in-sample or in-column conversion. Additionally, pteroic acid (Pte) derivatives were observed as oxidative cleavage products formed after removal of glutamate residue from folate structure. These derivatives appeared at a different retention time than the compound peak, also indicating in-sample or in-column conversion. These degradation products provided common degradation paths for folate polyglutamates.

H_4_PteGlu displayed products involving the addition of one-carbon unit (CH, CH_2_ and CH_3_), which may arise from formaldehyde (CH_2_O) contamination. Previous studies have demonstrated the chemical conversion of H_4_PteGlu to CH_2_-H_4_PteGlu [4], and its subsequent reduction to CH_3_-H_4_PteGlu. These interconversion products, displaying the addition of one-carbon unit, have the same retention times as those expected for their respective standard compounds, indicating in-sample or in-column conversion.

Dissociation of one-carbon units from CH=H_4_PteGlu and CH_2_-H_4_PteGlu, resulting in H_4_PteGlu was observed. We observed this dissociation occurring in both acidic and basic eluents. This finding contrast with the previous study that CH=H_4_PteGlu would be converted to H_4_PteGlu in physiological and acidic pH [5]. Furthermore, CH=H_4_PteGlu was observed to undergo ring opening, followed by hydration, resulting in the formation of CHO-H_4_PteGlu. Our observation is consistent with previous reports of interconversion between CH=H_4_PteGlu, 5-CHO-H_4_PteGlu, and 10-CHO-H_4_PteGlu [6, 7].

**Supplementary Note 2. Quality of LC separation of folate polyglutamates**

The quality of chromatographic separation was evaluated by peak resolution and width. We focused on quantifying peak resolution of adjacent peak pairs and folate redox couples that differ by 2 amu (i.e., PteGlu/H_2_PteGlu, H_2_PteGlu/H_4_PteGlu, CH=H_4_PteGlu/CH_2_-H_4_PteGlu, and CH_2_-H_4_PteGlu/CH_3_-H_4_PteGlu). Separating these pairs would resolve oxidative interconversion products and overlapping isotopic patterns. Separating folate polyglutamates is important for in-source CID and MS2 as folate species differing by the lengths of polyglutamate tails mainly produced the same fragment ion F_1_.

HILIC method I basic (pH 9.4) achieved limited separation with substantially overlapped peaks within a narrow retention time window (e.g., 14.59 to14.78 min for most monoglutamates) with no baseline separation (resolution factor *R_s_* = 0.17-0.62) (**Fig. S7**). To improve peak separation, we modified solvent gradient, resulting in 40-min HILIC method II. HILIC method II resolved redox pairs of CH=H_4_PteGlu/CH_2_-H_4_PteGlu (*R_s_* = 9) and CH_2_-H_4_PteGlu/CH_3_-H_4_PteGlu (*R_s_* = 1.7). The resolution of H_2_PteGlu/H_4_PteGlu pair showed slight improvements (*R_s_* = 0.61) compared to method I (*R_s_* = 0.27) (**Table S7**).

Folates retained slightly longer under acidic eluents (**Table S6** and **Fig. S7**) than under basic conditions. The last analyte peak appears at 19.59 min in HILIC method II.basic versus 20.83 min in HILIC method II.acidic. Despite some variations in separation between adjacent peak pairs for acidic versus basic HILIC methods, a substantial improvement was achieved with acidic eluent in HILIC method II as the baseline separation of H_2_PteGlu/H_4_PteGlu redox pair (*R_s_* = 1.77) (**Table S7**). Retention times in acidic and basic eluent conditions were correlated.

RPLC separated folate monoglutamates well with *R_s_*=7.39 for H_2_PteGlu and H_4_PteGlu. Compared to previously reported methods using ion-pairing agents [8, 9], our RPLC method offered a distinct advantage of allowing both positive and negative ion modes.

**Supplementary Figures**

**
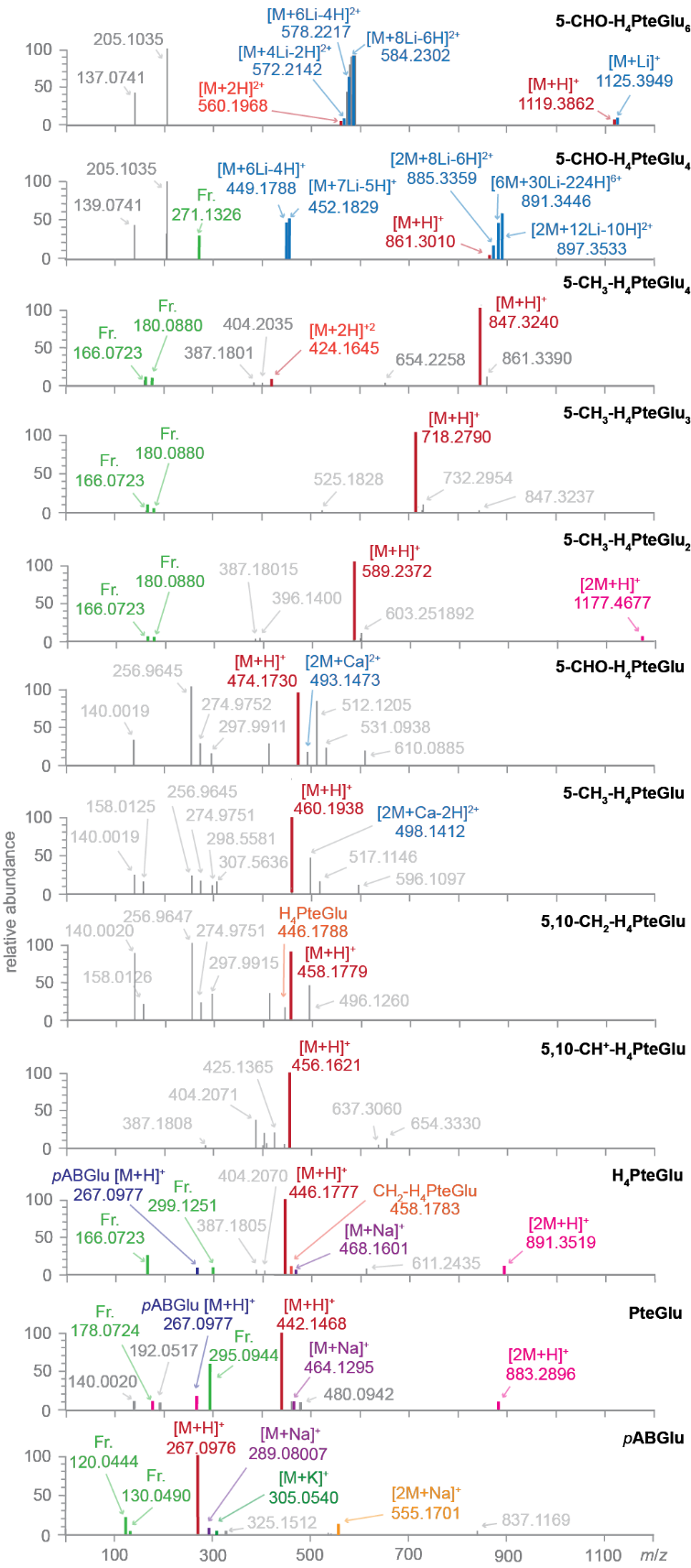
**

**Figure S1. Direct infusion mass spectrometry of folate standards in acidic eluents.** Mass spectra were collected in full scan positive ion mode. Precursor ion peaks are denoted in red, cationic adducts in blue, interconversion and degradation products in purple, dimer ions in pink, fragment ions in green, and unidentified peaks in grey.

**
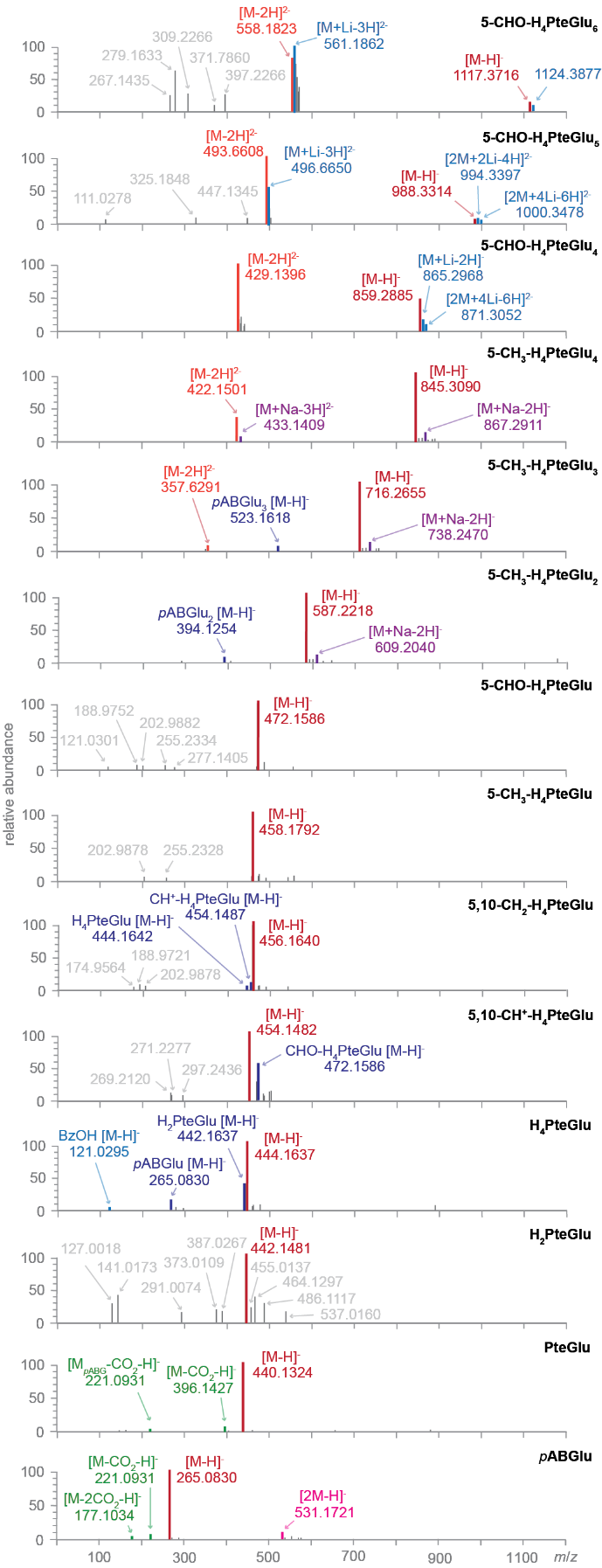
**

**Figure S2. Direct infusion mass spectrometry of folate standards in basic eluents.** Mass spectra were collected in full scan negative ion mode. Precursor ion peaks are denoted in red, cationic adducts in blue, interconversion and degradation products in purple, dimer ions in pink, fragment ions in green, and unidentified peaks in grey.

**
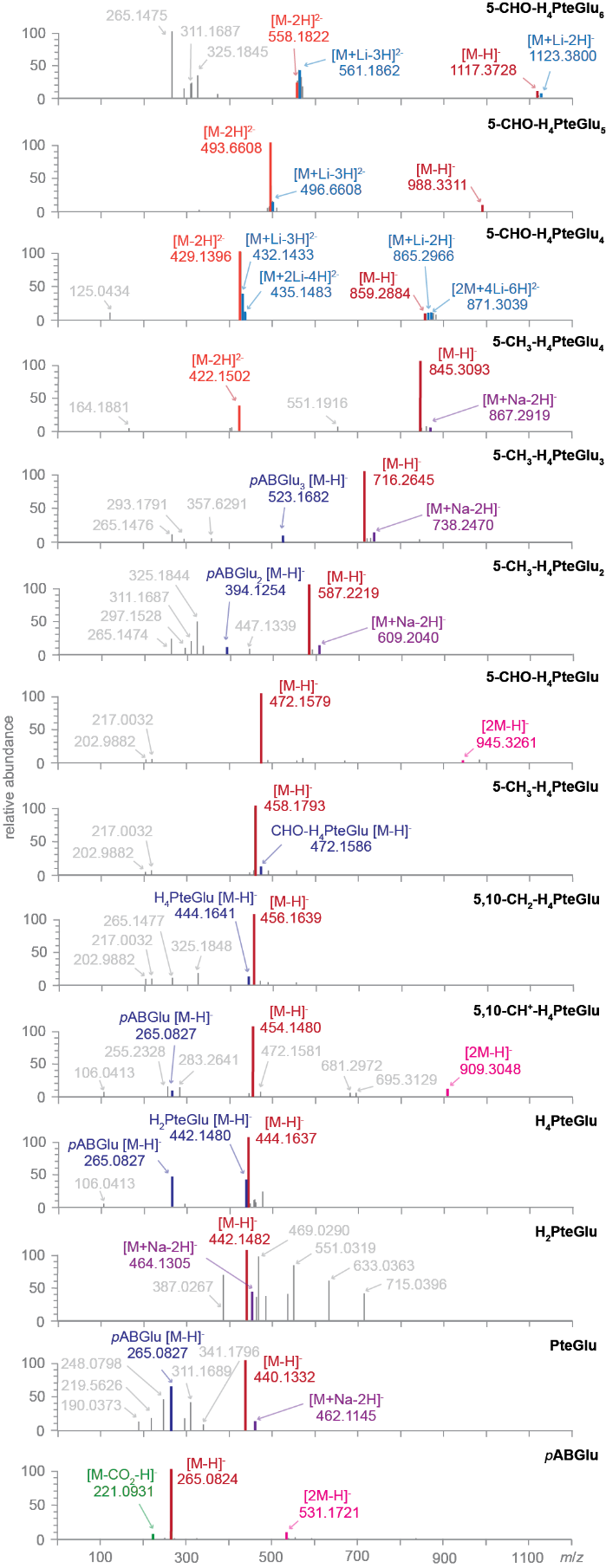
**

**Figure S3. Direct infusion mass spectrometry of folate standards in acidic eluents.** Mass spectra were collected in full scan negative ion mode. Precursor ion peaks are denoted in red, cationic adducts in blue, interconversion and degradation products in purple, dimer ions in pink, fragment ions in green, and unidentified peaks in grey.

**
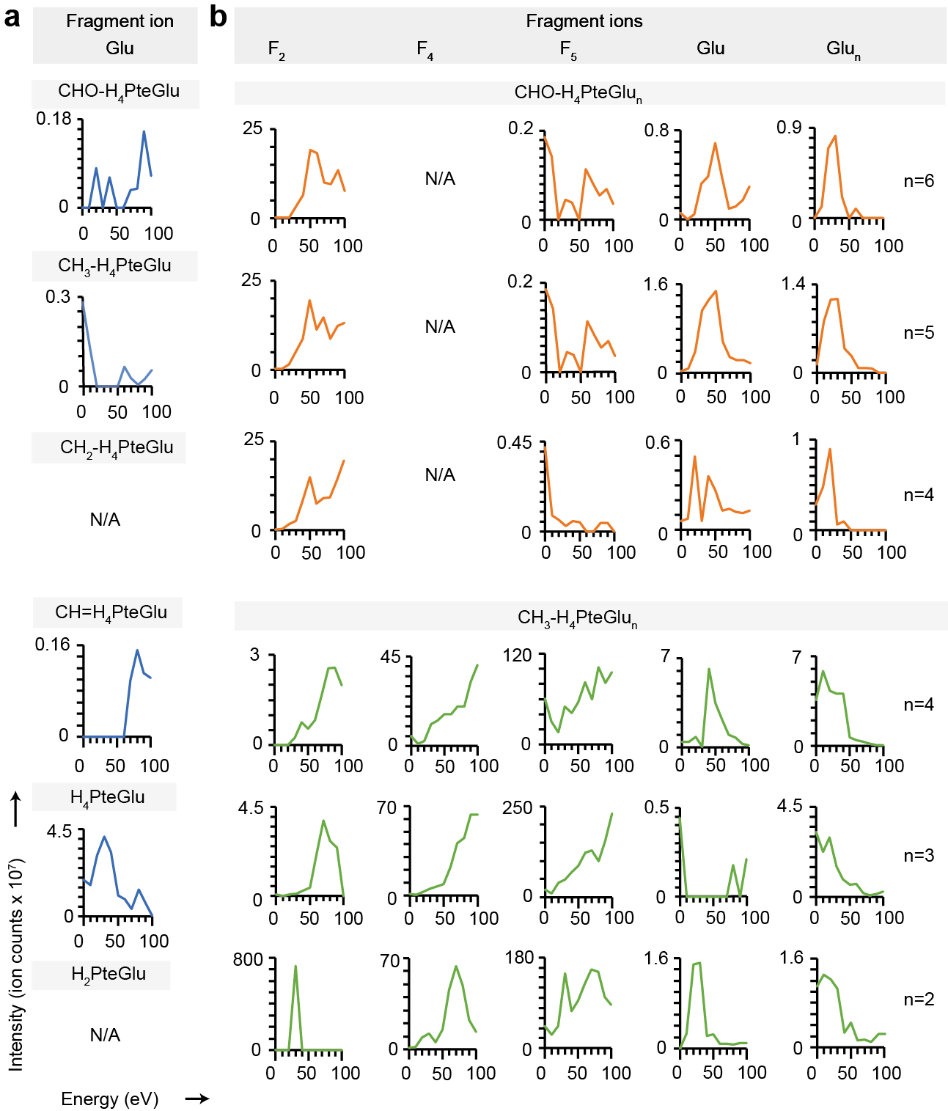
**

**Figure S4. Fragmentation products from in-source CID of folate polyglutamates.** **(a)** Glutamate was observed as a fragmentation product from in-source CID of folate monoglutamates. **(b)** Glutamate and polyglutamate as well as fragments F_2_, F_3_, and F_4_ were observed from in-source CID of folate polyglutamates.

**
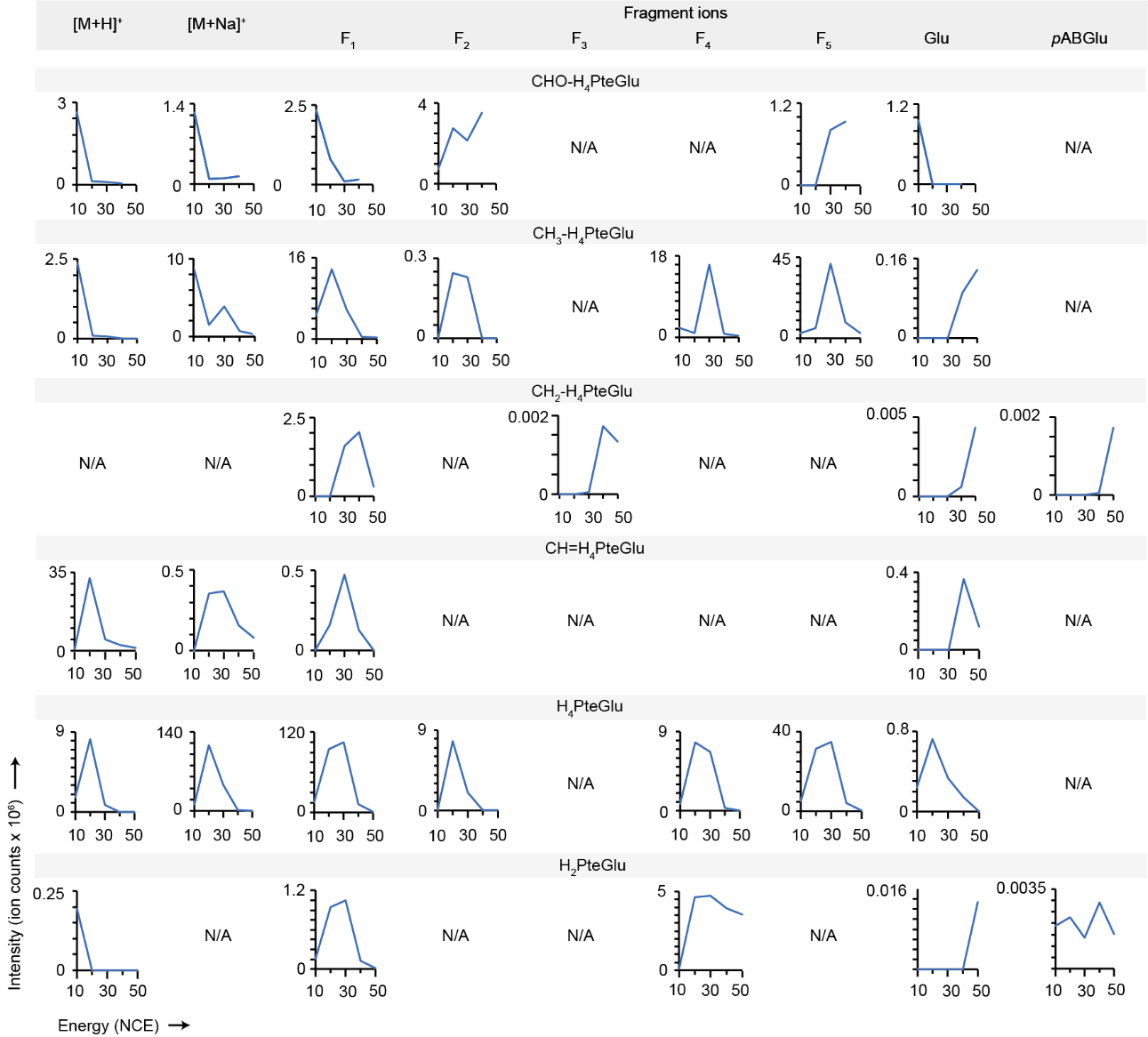
**

**Figure S5. Fragmentation products from AIF of folate monoglutamates on MS2.** Fragments F_1_ through F_5_ as well as glutamate and pABGlu were formed. F_3_ was unique to CH_2_-THF.


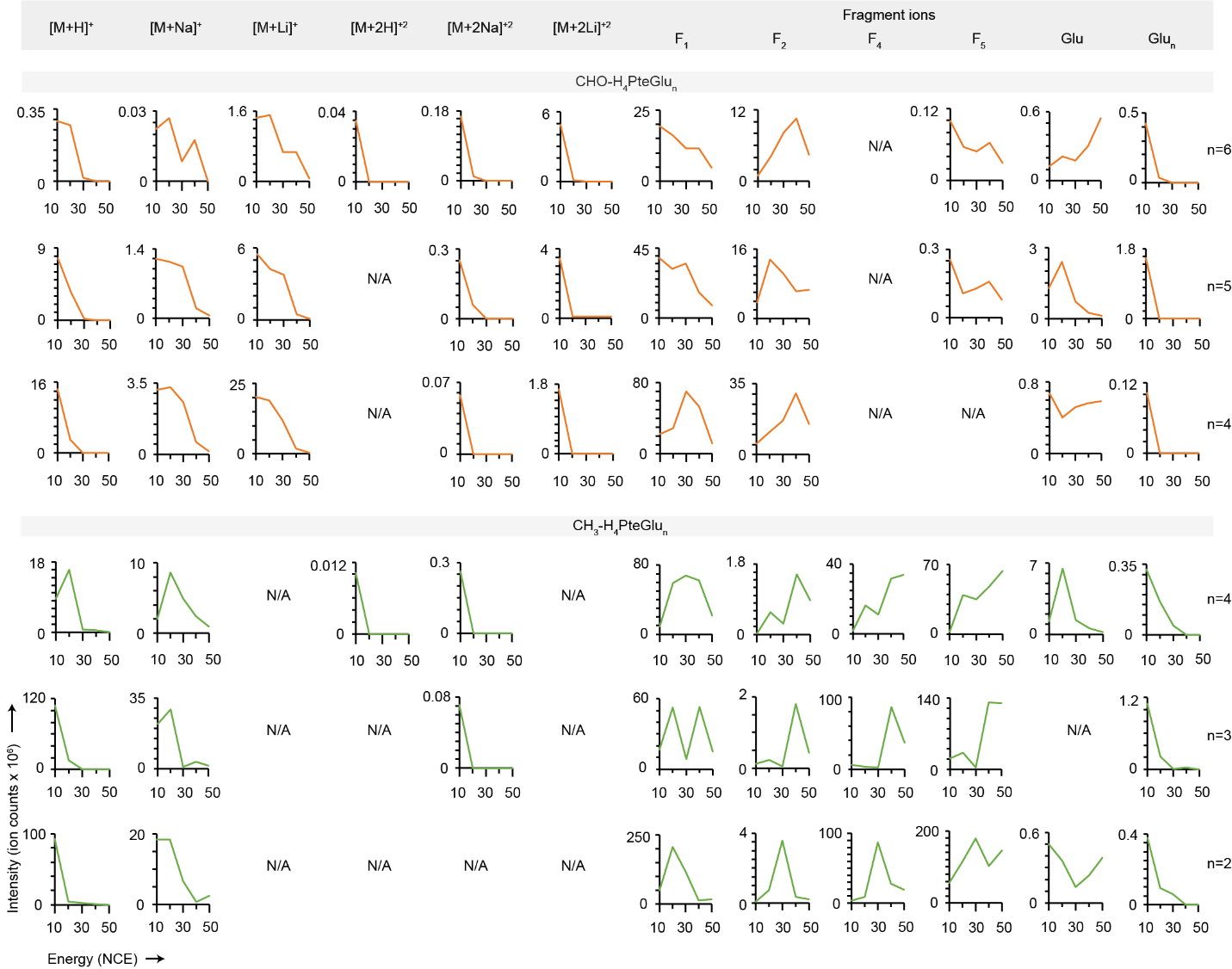


**Figure S6. Fragmentation products from AIF of folate polyglutamates on MS2.** Fragments F_1_, F_2_, F_4_, and F_5_ as well as glutamate and polyglutamates were formed.


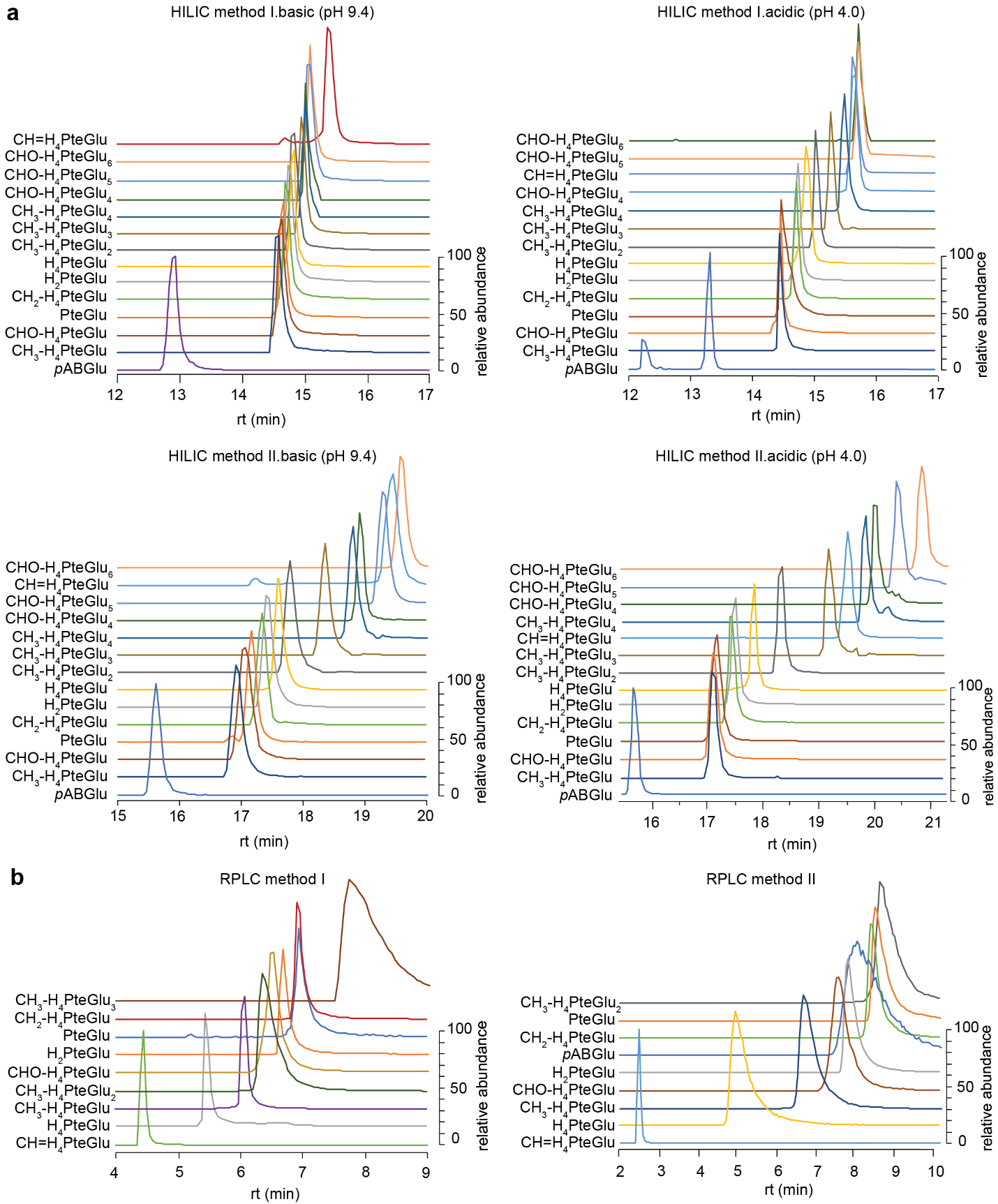


**Figure S7.** **LC separation of folate standards.** Extracted ion chromatograms (EICs) of folate standards were obtained from **(a)** HILIC or **(b)** RPLC coupled to MS operating in positive ion mode.

**
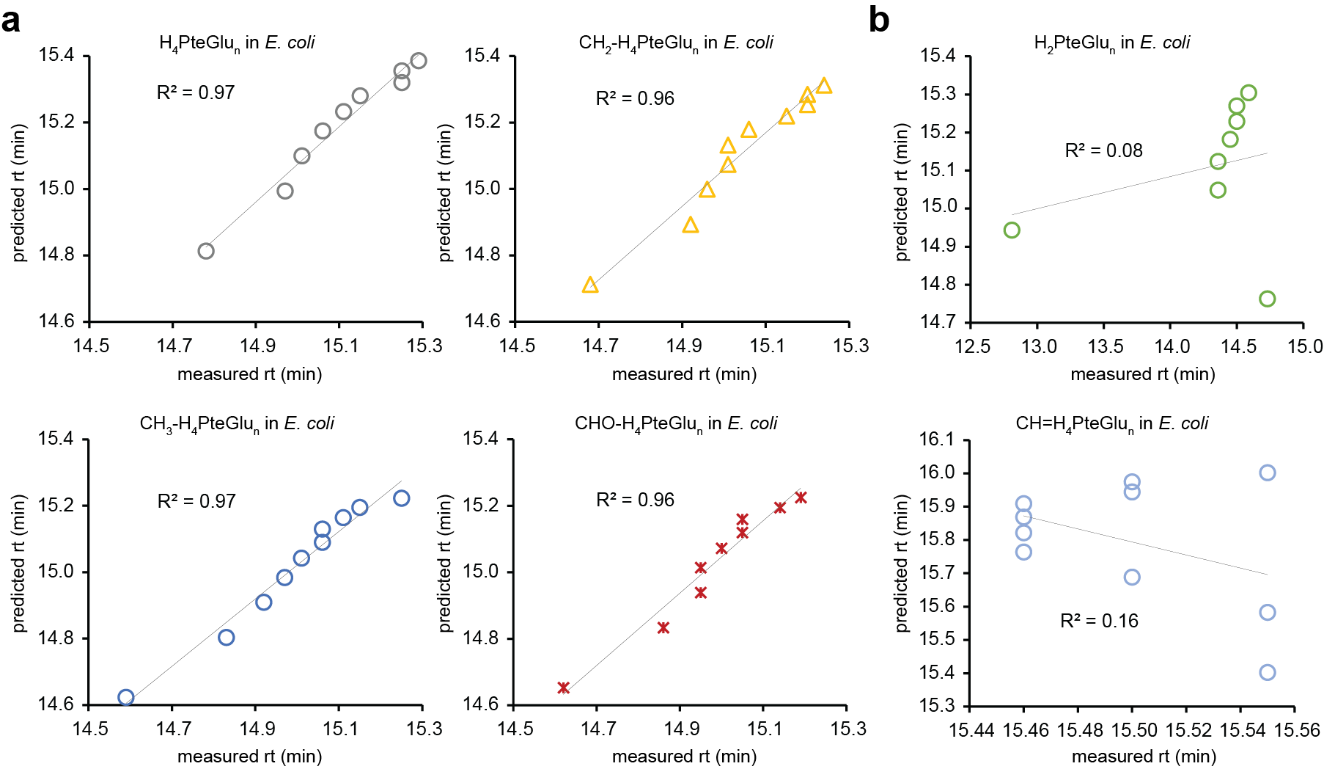
**

**Figure S8. Predicted versus observed retention times of folates in HILIC. (a)** The retention times of folate polyglutamates from *E. coli* in HILIC (method I basic) were measured and compared to predictions. **(b)** For CH=H_4_PteGlu and H_2_PteGlu, the lengths of polyglutamate tails did not alter retention times unlike other folate species, resulting in poor prediction of their retention times.

**Supplementary Tables**

**Table S1.** Previously studies on LC-MS-based determination of folates.

| **no.** | **detected folate derivatives** | **sample** | **LC mode** | **Ionization** | **MS analyzer** | **MS monitoring** | **ref.** |
| --- | --- | --- | --- | --- | --- | --- | --- |
| *Methods detected folate polyglutamates as is* | | | | | | | |
| 1 | PteGlu  H_4_PteGlu  CH=H_4_PteGlu  5-CH_3_-H_4_PteGlu_1-9_  10-CHO-H_4_PteGlu | *Saccharomyces cerevisiae* | RPLC | ESI (+) | QToF | DDA | [10] |
| 2 | H_2_PteGlu_1-5_  H_4_PteGlu_1-5_  CH=H_4_PteGlu_1-5_  CH_2_-H_4_PteGlu_1-5_  5-CH_3_-H_4_PteGlu_1-5_  10-CHO-H_4_PteGlu_1-5_ | *Escherichia coli* MG1655 | RPLC | ESI (-) | (Q)-Orbitrap | Full scan | [11] |
| 3 | PteGlu_1-6_  H_4_PteGlu_1-6_  CH=H_4_PteGlu_1-6_  CH_2_-H_4_PteGlu_1-6_  5-CH_3_-H_4_PteGlu_1-6_  5-CHO-H_4_PteGlu_1-6_ | Human liver cancer cell line (HepG2) | HILIC | ESI (+) | QqQ-LIT | MRM | [3] |
| 4 | PteGlu_1-6_  H_4_PteGlu_1-6_  CH=H_4_PteGlu_1-6_  CH_2_-H_4_PteGlu_1-6_  5-CH_3_-H_4_PteGlu_1-6_  5-CHO-H_4_PteGlu_1-6_ | *E. coli* K12 | HILIC | ESI (+) | QIT | MRM | [3] |
| 5 | PteGlu  H_4_PteGlu  CH=H_4_PteGlu  5-CH_3_-H_4_PteGlu_1-6_  5/10-CHO-H_4_PteGlu | Beans | RPLC | ESI (+) | QqQ | MRM | [12] |
| 6 | PteGlu  H_4_PteGlu  5-CH_3_-H_4_PteGlu_1-5_  5/10-CHO-H_4_PteGlu | *Bifidobacterium adolescentis* | RPLC | ESI (+) | QqQ | MRM | [13] |
| 7 | PteGlu_1-7_  H_4_PteGlu_1-11_  CH=H_4_PteGlu  5-CH_3_-H_4_PteGlu_1-11_  5-CHO-H_4_PteGlu_1-11_ | Erythrocytes | RPLC | ESI (+) | QqQ | SIM | [8] |
| 8 | 5-CH_3_-H_4_PteGlu_1-8_ | Vegetables | RPLC | ESI (+) | QqQ | SRM | [14] |
| 9 | pABGlu_1-5_  PteGlu_1-5_  H_2_PteGlu_1-5_  H_4_PteGlu_1-5_  CH=H_4_PteGlu_1-5_  CH_2_-H_4_PteGlu_1-5_  5-CH_3_-H_4_PteGlu_1-6_  5/10-CHO-H_4_PteGlu_1-5_ | *E. coli* K12 NCM3722 | HILIC | ESI (+)/(-) | QqQ | MRM | [1, 2] |
| 10 | pABGlu_2_  5-CH_3_-H_4_PteGlu_1-7_  5/10-CHO-H_4_PteGlu_1-2_ | *S. cerevisiae* | HILIC | ESI (+)/(-) | QqQ | MRM | [1, 2] |
| 11 | PteGlu_1-8_  H_2_PteGlu_1-8_  H_4_PteGlu_1-8_  CH=H_4_PteGlu_1-8_  5-CH_3_-H_4_PteGlu_1-8_  5/10-CHO-H_4_PteGlu_1-8_ | Plant and animal tissue | RPLC | ESI (-) | QqQ | MRM | [9] |
| 12 | PteGlu_1-7_  H_2_PteGlu_1-7_  H_4_PteGlu_1-7_  CH=H_4_PteGlu  CH_2_-H_4_PteGlu_1-7_  5-CH_3_-H_4_PteGlu_1-7_  5-CHO-H_4_PteGlu | *E. coli* OP50  *E. coli* HT1115  Lymphocytes  Human/mouse fibroblast  Mouse embryo | RPLC | ESI (-) | QqQ | MRM | [15] |
| *Methods detected folate polyglutamates after cleavage* | | | | | | | |
| 13 | H_2_PteGlu  H_4_PteGlu  CH=H_4_PteGlu  CH_2_-H_4_PteGlu  5-CH_3_-H_4_PteGlu  10-CHO-H_4_PteGlu | Human kidney cell line (293T)  Human colon cancer cell line (HCT116)  Human pancreas cancer cell line (8988T)  Human liver cancer cell line (HepG2) | RPLC | ESI-(+)/(-) | (Q)-Orbitrap | Full scan | [16] |
| *Methods for folate monoglutamates* | | | | | | | |
| 14 | PteGlu  H_2_PteGlu  H_4_PteGlu  CH=H_4_PteGlu  CH_2_-H_4_PteGlu  5-CH_3_-H_4_PteGlu  5-CHO-H_4_PteGlu | *Caenorhabditis*  *elegans* | RPLC | ESI/APCI-(+) | QqQ |  | [17] |
| 15 | PteGlu  H_2_PteGlu  H_4_PteGlu  CH=H_4_PteGlu  CH_2_-H_4_PteGlu  5-CH_3_-H_4_PteGlu  5-CHO-H_4_PteGlu | Pulse seed | RPLC | ESI-(+) | QqQ | SRM | [18] |
| 16 | PteGlu  H_4_PteGlu  CH=H_4_PteGlu  CH_2_-H_4_PteGlu  5-CH_3_-H_4_PteGlu  5-CHO-H_4_PteGlu | Tissue  Blood  Plasma | RPLC | ESI-(+) | QqQ | MRM | [19] |
| 17 | PteGlu  5-CH_3_-H_4_PteGlu | Plasma | RPLC | ESI | QqQ | MRM | [20] |
| 18 | PteGlu  H_4_PteGlu  5-CH_3_-H_4_PteGlu  5-CHO-H_4_PteGlu | Vegetables  Meat  Cereals | RPLC | ESI-(+) | IT | SRM | [21] |

Abbreviations: RPLC, reversed phase liquid chromatography; HILIC, hydrophilic interactions liquid chromatography; ESI, electrospray ionization; QqQ, triple quadrupole; QIT, quadrupole ion trap; IT, ion trap; TOF, time of flight; DDA, data independent acquisition; MRM, multiple reaction monitoring; SRM, single reaction monitoring. *p*ABGlu for *p*-aminobenzoyl-glutamate, PteGlu for pteroylglutamate (folate); H_4_PteGlu for tetrahydropteroylglutamate (tetrahydrofolate); H_2_PteGlu for dihydropteroylglutamate (dihydrofolate), 5/10-CHO-H_4_PteGlu for formyl-tetrahydropteroylglutamate, 5,10-CH=H_4_PteGlu for methenyl-tetrahydropteroylglutamate, 5,10-CH_2_-H_4_PteGlu for methylene-tetrahydropteroylglutamate, 5-CH_3_-H_4_PteGlu for methyl-tetrahydropteroylglutamate.

**Table S2**. Authenticated folate standards used in the present study.

| **compounds name** | **formula (as supplied)** | **molecular weight (as supplied)** | **purity (%)** | **abbreviation** |
| --- | --- | --- | --- | --- |
| 7,8-dihydrofolic acid | C_19_H_21_N_7_O_6_ | 443.4 | 93.0 | H_2_PteGlu |
| (6*S*)-5,6,7,8-tetrahydrofolic acid | C_19_H_23_N_7_O_6_ | 445.4 | 98.0 | H_4_PteGlu |
| (6*R*, *S*)-5,10-methenyl-5,6,7,8-tetrahydrofolic acid chloride | C_20_H_22_N_7_O_6_(Cl) | 491.9 | 97.2 | 5,10-CH=H_4_PteGlu |
| (6*R*, *S*)-5,10-methylene-5,6,7,8-tetrahydrofolic acid, calcium salt | C_20_H_23_N_7_O_6_(Ca) | 495.5 | 95.1 | 5,10-CH_2_-H_4_PteGlu |
| (6*R*, *S*)-5-methyl-5,6,7,8-tetrahydrofolic acid, calcium salt | C_20_H_23_N_7_O_6_•4H_2_O(Ca) | 497.5 | 98.4 | 5-CH_3_-H_4_PteGlu |
| (6*S*)-5-formyl-5,6,7,8-tetrahydrofolic acid, calcium salt | C_20_H_21_N_7_O_7_(Ca) | 511.5 | 99.6 | 5-CHO-H_4_PteGlu |
| (6*R*, *S*)-5-methyl-5,6,7,8-tetrahydropteroyldi-*γ*-*L*-glutamic acid trihydrochloride | C_25_H_32_N_8_O_9_•3HCl | 698 | 93.8 | 5-CH_3_-H_4_PteGlu_2_ |
| (6*R*, *S*)-5-methyl-5,6,7,8-tetrahydropteroyltri-*γ*-*L*-glutamic acid trihydrochloride | C_30_H_39_N_9_O_12_•3HCl | 827.1 | 93.0 | 5-CH_3_-H_4_PteGlu_3_ |
| (6*R, S*)-5-methyl-5,6,7,8-tetrahydropteroyltetra-*γ*-*L*-glutamic acid trihydrochloride | C_35_H_46_N_10_O_15_•3HCl | 956.2 | 90.1 | 5-CH_3_-H_4_PteGlu_4_ |
| (6*R*, *S*)-5-formyl-5,6,7,8-tetrahydropteroyltetra-*γ*-*L*-glutamic acid, lithium salt | C_35_H_39_N_10_O_16_(Li)_5_ | 890.5 | 95.7 | 5-CHO-H_4_PteGlu_4_ |
| (6*R*, *S*)-5-formyl-5,6,7,8-tetrahydropteroylpenta-*γ*-*L*-glutamic acid, lithium salt | C_40_H_45_N_11_O_16_(Li)_6_ | 1025.5 | 97.7 | 5-CHO-H_4_PteGlu_5_ |
| (6*R*, *S*)-5-formyl-5,6,7,8-tetrahydropteroylhexa-*γ*-*L*-glutamic acid, lithium salt | C_45_H_51_N_12_O_22_(Li)_7_ | 1160.6 | 98.8 | 5-CHO-H_4_PteGlu_6_ |

**Table S3**. Chemical formulae for folate polyglutamates

| **compound** | **abbreviation** | **formula** |
| --- | --- | --- |
| glutamate | Glu | C_5_H_9_NO_4_ |
| diglutamate | Glu_2_ | C_10_H_16_N_2_O_7_ |
| triglutamate | Glu_3_ | C_15_H_23_N_3_O_10_ |
| tetraglutamate | Glu_4_ | C_20_H_30_N_4_O_13_ |
| pentaglutamate | Glu_5_ | C_25_H_37_N_5_O_16_ |
| hexaglutamate | Glu_6_ | C_30_H_44_N_6_O_19_ |
| heptaglutamate | Glu_7_ | C_35_H_51_N_7_O_22_ |
| octaglutamate | Glu_8_ | C_40_H_58_N_8_O_25_ |
| nonaglutamate | Glu_9_ | C_45_H_65_N_9_O_28_ |
| decaglutamate | Glu_10_ | C_50_H_72_N_10_O_31_ |
| *para*-aminobenzoylglutamate | *p*ABGlu | C_12_H_14_N_2_O_5_ |
| *para*-aminobenzoyldiglutamate | *p*ABGlu_2_ | C_17_H_21_N_3_O_8_ |
| *para*-aminobenzoyltriglutamate | *p*ABGlu_3_ | C_22_H_28_N_4_O_11_ |
| *para*-aminobenzoyltetraglutamate | *p*ABGlu_4_ | C_27_H_35_N_5_O_14_ |
| *para*-aminobenzoylpentadiglutamate | *p*ABGlu_5_ | C_32_H_42_N_6_O_17_ |
| *para*-aminobenzoylhexaglutamate | *p*ABGlu_6_ | C_37_H_49_N_7_O_20_ |
| *para*-aminobenzoylheptaglutamate | *p*ABGlu_7_ | C_42_H_56_N_8_O_23_ |
| *para*-aminobenzoyloctaglutamate | *p*ABGlu_8_ | C_47_H_63_N_9_O_26_ |
| *para*-aminobenzoylnonaglutamate | *p*ABGlu_9_ | C_52_H_70_N_10_O_29_ |
| *para*-aminobenzoyldecaglutamate | *p*ABGlu_10_ | C_57_H_77_N_11_O_32_ |
| pteroylglutamate | PteGlu | C_19_H_19_N_7_O_6_ |
| pteroyldiglutamate | PteGlu_2_ | C_24_H_26_N_8_O_9_ |
| pteroyltriglutamate | PteGlu_3_ | C_29_H_33_N_9_O_12_ |
| pteroyltetraglutamate | PteGlu_4_ | C_34_H_40_N_10_O_15_ |
| pteroylpentaglutamate | PteGlu_5_ | C_39_H_47_N_11_O_18_ |
| pteroylhexaglutamate | PteGlu_6_ | C_44_H_54_N_12_O_21_ |
| pteroylheptaglutamate | PteGlu_7_ | C_49_H_61_N_13_O_24_ |
| pteroyloctaglutamate | PteGlu_8_ | C_54_H_68_N_14_O_27_ |
| pteroylnonaglutamate | PteGlu_9_ | C_59_H_75_N_15_O_30_ |
| pteroyldecaglutamate | PteGlu_10_ | C_64_H_82_N_16_O_33_ |
| dihydropteroylglutamate | H_2_PteGlu | C_19_H_21_N_7_O_6_ |
| dihydropteroyldiglutamate | H_2_PteGlu_2_ | C_24_H_28_N_8_O_9_ |
| dihydropteroyltriglutamate | H_2_PteGlu_3_ | C_29_H_35_N_9_O_12_ |
| dihydropteroyltetraglutamate | H_2_PteGlu_4_ | C_34_H_42_N_10_O_15_ |
| dihydropteroylpentaglutamate | H_2_PteGlu_5_ | C_39_H_49_N_11_O_18_ |
| dihydropteroylhexaglutamate | H_2_PteGlu_6_ | C_44_H_56_N_12_O_21_ |
| dihydropteroylheptaglutamate | H_2_PteGlu_7_ | C_49_H_63_N_13_O_24_ |
| dihydropteroyloctaglutamate | H_2_PteGlu_8_ | C_54_H_70_N_14_O_27_ |
| dihydropteroylnonaglutamate | H_2_PteGlu_9_ | C_59_H_77_N_15_O_30_ |
| dihydropteroyldecaglutamate | H_2_PteGlu_10_ | C_64_H_84_N_16_O_33_ |
| tetrahydropteroylglutamate | H_4_PteGlu | C_19_H_23_N_7_O_6_ |
| tetrahydropteroyldiglutamate | H_4_PteGlu_2_ | C_24_H_30_N_8_O_9_ |
| tetrahydropteroyltriglutamate | H_4_PteGlu_3_ | C_29_H_37_N_9_O_12_ |
| tetrahydropteroyltetraglutamate | H_4_PteGlu_4_ | C_34_H_44_N_10_O_15_ |
| tetrahydropteroylpentaglutamate | H_4_PteGlu_5_ | C_39_H_51_N_11_O_18_ |
| tetrahydropteroylhexaglutamate | H_4_PteGlu_6_ | C_44_H_58_N_12_O_21_ |
| tetrahydropteroylheptaglutamate | H_4_PteGlu_7_ | C_49_H_65_N_13_O_24_ |
| tetrahydropteroyloctaglutamate | H_4_PteGlu_8_ | C_54_H_72_N_14_O_27_ |
| tetrahydropteroylnonaglutamate | H_4_PteGlu_9_ | C_59_H_79_N_15_O_30_ |
| tetrahydropteroyldecaglutamate | H_4_PteGlu_10_ | C_64_H_86_N_16_O_33_ |
| methenyltetrahydropteroylglutamate | CH=H_4_PteGlu | C_20_H_21_N_7_O_6_ |
| methenyltetrahydropteroyldiglutamate | CH=H_4_PteGlu_2_ | C_25_H_28_N_8_O_9_ |
| methenyltetrahydropteroyltriglutamate | CH=H_4_PteGlu_3_ | C_30_H_35_N_9_O_12_ |
| methenyltetrahydropteroyltetraglutamate | CH=H_4_PteGlu_4_ | C_35_H_42_N_10_O_15_ |
| methenyltetrahydropteroylpentaglutamate | CH=H_4_PteGlu_5_ | C_40_H_49_N_11_O_18_ |
| methenyltetrahydropteroylhexaglutamate | CH=H_4_PteGlu_6_ | C_45_H_56_N_12_O_21_ |
| methenyltetrahydropteroylheptaglutamate | CH=H_4_PteGlu_7_ | C_50_H_63_N_13_O_24_ |
| methenyltetrahydropteroyloctaglutamate | CH=H_4_PteGlu_8_ | C_55_H_70_N_14_O_27_ |
| methenyltetrahydropteroylnonaglutamate | CH=H_4_PteGlu_9_ | C_60_H_77_N_15_O_30_ |
| methenyltetrahydropteroyldecaglutamate | CH=H_4_PteGlu_10_ | C_65_H_84_N_16_O_33_ |
| methylenetetrahydropteroylglutamate | CH_2_-H_4_PteGlu | C_20_H_23_N_7_O_6_ |
| methylenetetrahydropteroyldiglutamate | CH_2_-H_4_PteGlu_2_ | C_25_H_30_N_8_O_9_ |
| methylenetetrahydropteroyltriglutamate | CH_2_-H_4_PteGlu_3_ | C_30_H_37_N_9_O_12_ |
| methylenetetrahydropteroyltetraglutamate | CH_2_-H_4_PteGlu_4_ | C_35_H_44_N_10_O_15_ |
| methylenetetrahydropteroylpentaglutamate | CH_2_-H_4_PteGlu_5_ | C_40_H_51_N_11_O_18_ |
| methylenetetrahydropteroylhexaglutamate | CH_2_-H_4_PteGlu_6_ | C_45_H_58_N_12_O_21_ |
| methylenetetrahydropteroylheptaglutamate | CH_2_-H_4_PteGlu_7_ | C_50_H_65_N_13_O_24_ |
| methylenetetrahydropteroyloctaglutamate | CH_2_-H_4_PteGlu_8_ | C_55_H_72_N_14_O_27_ |
| methylenetetrahydropteroylnonaglutamate | CH_2_-H_4_PteGlu_9_ | C_60_H_79_N_15_O_30_ |
| methylenetetrahydropteroyldecaglutamate | CH_2_-H_4_PteGlu_10_ | C_65_H_86_N_16_O_33_ |
| methyltetrahydropteroylglutamate | CH_3_-H_4_PteGlu | C_20_H_25_N_7_O_6_ |
| methyltetrahydropteroyldiglutamate | CH_3_-H_4_PteGlu_2_ | C_25_H_32_N_8_O_9_ |
| methyltetrahydropteroyltriglutamate | CH_3_-H_4_PteGlu_3_ | C_30_H_39_N_9_O_12_ |
| methyltetrahydropteroyltetraglutamate | CH_3_-H_4_PteGlu_4_ | C_35_H_46_N_10_O_15_ |
| methyltetrahydropteroylpentaglutamate | CH_3_-H_4_PteGlu_5_ | C_40_H_53_N_11_O_18_ |
| methyltetrahydropteroylhexaglutamate | CH_3_-H_4_PteGlu_6_ | C_45_H_60_N_12_O_21_ |
| methyltetrahydropteroylheptaglutamate | CH_3_-H_4_PteGlu_7_ | C_50_H_67_N_13_O_24_ |
| methyltetrahydropteroyloctaglutamate | CH_3_-H_4_PteGlu_8_ | C_55_H_74_N_14_O_27_ |
| methyltetrahydropteroylnonaglutamate | CH_3_-H_4_PteGlu_9_ | C_60_H_81_N_15_O_30_ |
| methyltetrahydropteroyldecaglutamate | CH_3_-H_4_PteGlu_10_ | C_65_H_88_N_16_O_33_ |
| formyltetrahydropteroylglutamate | CHO-H_4_PteGlu | C_20_H_23_N_7_O_7_ |
| formyltetrahydropteroyldiglutamate | CHO-H_4_PteGlu_2_ | C_25_H_30_N_8_O_10_ |
| formyltetrahydropteroyltriglutamate | CHO-H_4_PteGlu_3_ | C_30_H_37_N_9_O_13_ |
| formyltetrahydropteroyltetraglutamate | CHO-H_4_PteGlu_4_ | C_35_H_44_N_10_O_16_ |
| formyltetrahydropteroylpentaglutamate | CHO-H_4_PteGlu_5_ | C_40_H_51_N_11_O_19_ |
| formyltetrahydropteroylhexaglutamate | CHO-H_4_PteGlu_6_ | C_45_H_58_N_12_O_22_ |
| formyltetrahydropteroylheptaglutamate | CHO-H_4_PteGlu_7_ | C_50_H_65_N_13_O_25_ |
| formyltetrahydropteroyloctaglutamate | CHO-H_4_PteGlu_8_ | C_55_H_72_N_14_O_28_ |
| formyltetrahydropteroylnonaglutamate | CHO-H_4_PteGlu_9_ | C_60_H_79_N_15_O_31_ |
| formyltetrahydropteroyldecaglutamate | CHO-H_4_PteGlu_10_ | C_65_H_86_N_16_O_34_ |

^1^pteroylglutamate, folate; dihydropteroylglutamate, dihydrofolate; tetrahydropteroylglutamate, tetrahydrofolate; methenyltetrahydropteroylglutamate, methenyltetrahydrofolate; methylenetetrahydropteroylglutamate, methylenetetrahydrofolate; methyltetrahydropteroylglutamate, methyltetrahydrofolate; formyltetrahydropteroylglutamate, formyltetrahydrofolate

**Table S4.** Response factors of folate polyglutamates (100 uM concentration) obtained by direct injection of standard compounds diluted in basic A in positive mode.

| **Analyte** | **C (uM)** | ***m/z*** | **Intensity ^a^** | **Response factor (Intensity/C)** | **Intensity relative to the respective monoglutamate form** |
| --- | --- | --- | --- | --- | --- |
| Positive ion mode | | | | | |
| CH_3_-H_4_PteGlu | 100 | 460.1933 | 6.E+06 | 6.E+04 | 1.00 |
| CH_3_-H_4_PteGlu_2_ | 100 | 589.2349 | 1.E+07 | 1.E+05 | 1.80 |
| CH_3_-H_4_PteGlu_3_ | 100 | 718.2790 | 6.E+06 | 6.E+04 | 0.90 |
| CH_3_-H_4_PteGlu_4_ | 100 | 847.3208 | 9.E+05 | 9.E+03 | 0.14 |
| CHO-H_4_PteGlu | 100 | 474.1720 | 5.E+06 | 5.E+04 | 1.00 |
| CHO-H_4_PteGlu_4_ | 100 | 861.3014 | 2.E+06 | 2.E+04 | 0.38 |
| CHO-H_4_PteGlu_5_ | 100 | 990.3404 | 9.E+05 | 9.E+03 | 0.20 |
| CHO-H_4_PteGlu_6_ | 100 | 1119.3861 | 3.E+05 | 3.E+03 | 0.06 |
| Negative ion mode | | | | | |
| CH_3_-H_4_PteGlu | 100 | 458.1796 | 1.08E+07 | 1.E+05 | 1.00 |
| CH_3_-H_4_PteGlu_2_ | 100 | 587.2221 | 2.72E+06 | 3.E+04 | 0.25 |
| CH_3_-H_4_PteGlu_3_ | 100 | 716.2657 | 1.72E+06 | 2.E+04 | 0.16 |
| CH_3_-H_4_PteGlu_4_ | 100 | 845.3093 | 3.95E+05 | 4.E+03 | 0.04 |
| CHO-H_4_PteGlu | 100 | 472.1583 | 4.56E+06 | 5.E+04 | 1.00 |
| CHO-H_4_PteGlu_4_ | 100 | 859.2887 | 6.08E+06 | 6.E+04 | 1.33 |
| CHO-H_4_PteGlu_5_ | 100 | 988.3318 | 1.39E+06 | 1.E+04 | 0.31 |
| CHO-H_4_PteGlu_6_ | 100 | 1117.3737 | 2.95E+05 | 3.E+03 | 0.06 |

^a^ intensity represents the average of triplicates

**Table S5.** Fragment ions of folate species formed during in-source fragmentation as well as in-source CID and AIF MS2 analysis in positive ion mode. Fragment ions F_1_ through F_5_ correspond to the structures illustrated in Fig. 4A.

| **Compound** | ***m/z*** | | | | |
| --- | --- | --- | --- | --- | --- |
|  | **F_1_** | **F_2_** | **F_3_** | **F_4_** | **F_5_** |
| H_2_PteGlu | 297.1094 | 269.1145 | 193.0832 | 178.0723 | 164.0567 |
| H_4_PteGlu | 299.1251 | 271.1302 | 195.0989 | 180.0880 | 166.0723 |
| CH=H_4_PteGlu | 309.1095 | 295.1302 | 205.0832 | N/A | N/A |
| CH_2_-H_4_PteGlu | 311.1251 | 297.1458 | 207.0989 | N/A | N/A |
| CH_3_-H_4_PteGlu | 313.1408 | 285.1458 | 209.1145 | 194.1036 | 180.0880 |
| 5-CHO-H_4_PteGlu | 327.1200 | 299.1251 | 223.0938 | 208.0829 | 194.0673 |
| 10-CHO-H_4_PteGlu | 327.1200 | 299.1251 | 195.0989 | 180.0880 | 166.0723 |

**Table S6**. LC retention times of folate standards.

| **Compound** | **rt (min)** | | | | | |
| --- | --- | --- | --- | --- | --- | --- |
|  | **HILIC method I.acidic** | **HILIC method I.basic** | **HILIC method II.acidic** | **HILIC method II.basic** | **RPLC method I** | **RPLC method II** |
| *p*ABGlu | 13.34 | 12.93 | 15.67 | 15.6 | N/A ^a^ | 8.40 |
| PteGlu | 14.47 | 14.65 | 17.1 | 17.17 | 6.92 | 8.87 |
| H_2_PteGlu | 14.74 | 14.73 | 17.5 | 17.42 | 6.67 | 8.13 |
| H_4_PteGlu | 14.88 | 14.78 | 17.83 | 17.58 | 5.42 | 5.20 |
| 5,10-CH=H_4_PteGlu | 15.68 | 15.37 | 19.52 | 19.47 | 4.43 | 2.64 |
| 5,10-CH_2_-H_4_PteGlu | 14.71 | 14.68 | 17.45 | 17.33 | 6.89 | 8.73 |
| 5-CH_3_-H_4_PteGlu | 14.44 | 14.59 | 17.08 | 16.91 | 6.05 | 6.96 |
| 5-CHO-H_4_PteGlu | 14.46 | 14.62 | 17.17 | 17.05 | 6.53 | 7.91 |
| 5-CH_3_-H_4_PteGlu_2_ | 15.03 | 14.83 | 18.33 | 17.78 | 6.35 | 8.95 |
| 5-CH_3_-H_4_PteGlu_3_ | 15.28 | 14.94 | 19.17 | 18.36 | 7.75 | N/A ^b^ |
| 5-CH_3_-H_4_PteGlu_4_ | 15.49 | 15.00 | 19.84 | 18.81 | N/A ^b^ | N/A ^b^ |
| 5-CHO-H_4_PteGlu_4_ | 15.56 | 15.05 | 20.09 | 18.93 | N/A ^b^ | N/A ^b^ |
| 5-CHO-H_4_PteGlu_5_ | 15.62 | 15.08 | 20.38 | 19.3 | N/A ^b^ | N/A ^b^ |
| 5-CHO-H_4_PteGlu_6_ | 15.72 | 15.07 | 20.83 | 19.59 | N/A ^b^ | N/A ^b^ |

^a^ Not tested. ^b^ Tested but no peak observed.

**Table S7**. LC peak resolution (Rs)

| **Analyte**  **(in elution order)** | **rt (min)** | **h_max_**  **(intensity)** | **h_0.5_**  **(intensity)** | **t_start_ @ h_0.5_ (min)** | **t_end_ @ h_0.5_ (min)** | **W_0.5_ (min)** | **Rs-adjacent band pairs** |  | **critical redox pairs** | **Rs** |
| --- | --- | --- | --- | --- | --- | --- | --- | --- | --- | --- |
| **HILIC method I.basic** | | | | | | | | | | |
| CH_3_-H_4_PteGlu | 14.59 | 9.88E+08 | 4.94E+08 | 14.54 | 14.64 | 0.10 | 0.17 |  | PteGlu | 0.43 |
| CHO-H_4_PteGlu | 14.62 | 6.42E+08 | 3.21E+08 | 14.57 | 14.68 | 0.11 | 0.17 |  | H_2_PteGlu |  |
| PteGlu | 14.65 | 1.99E+08 | 9.93E+07 | 14.60 | 14.70 | 0.10 | 0.24 |  |  |  |
| CH_2_-H_4_PteGlu | 14.68 | 3.85E+08 | 1.93E+08 | 14.68 | 14.73 | 0.05 | 0.35 |  | H_2_PteGlu | 0.27 |
| H_2_PteGlu | 14.73 | 2.56E+08 | 1.28E+08 | 14.68 | 14.80 | 0.12 | 0.27 |  | H_4_PteGlu |  |
| H_4_PteGlu | 14.78 | 1.82E+08 | 9.11E+07 | 14.76 | 14.86 | 0.10 | 0.30 |  |  |  |
| CH_3_-H_4_PteGlu_2_ | 14.83 | 9.49E+08 | 4.75E+08 | 14.78 | 14.88 | 0.10 | 0.62 |  | CH_2_-H_4_PteGlu | -4.29 |
| CH_3_-H_4_PteGlu_3_ | 14.94 | 9.27E+08 | 4.63E+08 | 14.9 | 15.01 | 0.11 | 0.32 |  | CH=H_4_PteGlu |  |
| CH_3_-H_4_PteGlu_4_ | 15.00 | 8.45E+08 | 4.22E+08 | 14.95 | 15.06 | 0.11 | 0.37 |  |  |  |
| CHO-H_4_PteGlu_4_ | 15.05 | 2.55E+08 | 1.27E+08 | 15.14 | 15.19 | 0.05 | 0.21 |  | CH_3_-H_4_PteGlu | -0.71 |
| CHO-H_4_PteGlu_5_ | 15.08 | 4.08E+08 | 2.04E+08 | 15.02 | 15.14 | 0.12 | -0.05 |  | CH_2_-H_4_PteGlu |  |
| CHO-H_4_PteGlu_6_ | 15.07 | 3.36E+08 | 1.68E+08 | 15.04 | 15.15 | 0.11 | 1.42 |  |  |  |
| CH=H_4_PteGlu | 15.37 | 6.57E+08 | 3.28E+08 | 15.34 | 15.48 | 0.14 |  |  |  |  |
| **HILIC method II.basic** | | | | | | | | | | |
| CH_3_-H_4_PteGlu | 16.91 | 1.21E+09 | 6.07E+08 | 16.85 | 17.01 | 0.16 | 0.45 |  | PteGlu | 1.13 |
| CHO-H_4_PteGlu | 17.05 | 6.57E+08 | 3.29E+08 | 16.95 | 17.16 | 0.21 | 0.44 |  | H_2_PteGlu |  |
| PteGlu | 17.17 | 2.97E+08 | 1.48E+08 | 17.12 | 17.23 | 0.11 | 0.79 |  |  |  |
| CH_2_-H_4_PteGlu | 17.33 | 5.43E+08 | 2.71E+08 | 17.27 | 17.4 | 0.13 | 0.38 |  | H_2_PteGlu | 0.61 |
| H_2_PteGlu | 17.42 | 3.27E+08 | 1.63E+08 | 17.35 | 17.50 | 0.15 | 0.61 |  | H_4_PteGlu |  |
| H_4_PteGlu | 17.58 | 4.70E+08 | 2.35E+08 | 17.5 | 17.66 | 0.16 | 0.84 |  |  |  |
| CH_3_-H_4_PteGlu_2_ | 17.78 | 1.28E+09 | 6.38E+08 | 17.72 | 17.84 | 0.12 | 0.31 |  | CH_2_-H_4_PteGlu | -9.02 |
| CH_3_-H_4_PteGlu_3_ | 18.36 | 1.31E+09 | 6.55E+08 | 16.3 | 18.41 | 2.11 | 0.24 |  | CH=H_4_PteGlu |  |
| CH_3_-H_4_PteGlu_4_ | 18.81 | 1.11E+09 | 5.57E+08 | 18.76 | 18.87 | 0.11 | 0.46 |  |  |  |
| CHO-H_4_PteGlu_4_ | 18.93 | 7.93E+04 | 3.97E+04 | 19.82 | 19.02 | 0.20 | 1.25 |  | CH_3_-H_4_PteGlu | -1.71 |
| CHO-H_4_PteGlu_5_ | 19.30 | 4.54E+08 | 2.27E+08 | 19.25 | 19.40 | 0.15 | 1.10 |  | CH_2_-H_4_PteGlu |  |
| CHO-H_4_PteGlu_6_ | 19.59 | 4.04E+08 | 2.02E+08 | 19.53 | 19.69 | 0.16 |  |  |  |  |
| CH=H_4_PteGlu | 19.47 | 6.54E+08 | 3.27E+08 | 19.35 | 19.5 | 0.15 |  |  |  |  |
| **HILIC method I.acidic** | | | | | | | | | | |
| CH_3_-H_4_PteGlu | 14.44 | 2.66E+08 | 1.33E+08 | 14.43 | 14.49 | 0.06 | 0.12 |  | PteGlu | 2.28 |
| CHO-H_4_PteGlu | 14.46 | 5.27E+07 | 2.63E+07 | 14.45 | 14.59 | 0.14 | 0.07 |  | H_2_PteGlu |  |
| PteGlu | 14.47 | 7.95E+07 | 3.98E+07 | 14.43 | 14.47 | 0.04 | 2.18 |  |  |  |
| CH_2_-H_4_PteGlu | 14.71 | 9.94E+07 | 4.97E+07 | 14.66 | 14.75 | 0.09 | 0.19 |  | H_2_PteGlu | 0.92 |
| H_2_PteGlu | 14.74 | 9.13E+07 | 4.56E+07 | 14.69 | 14.79 | 0.10 | 0.92 |  | H_4_PteGlu |  |
| H_4_PteGlu | 14.88 | 3.56E+07 | 1.78E+07 | 14.87 | 14.95 | 0.08 | 1.36 |  |  |  |
| CH_3_-H_4_PteGlu_2_ | 15.03 | 4.81E+08 | 2.40E+08 | 15.03 | 15.08 | 0.05 | 2.95 |  | CH_2_-H_4_PteGlu | -6.02 |
| CH_3_-H_4_PteGlu_3_ | 15.28 | 4.89E+08 | 2.44E+08 | 15.28 | 15.33 | 0.05 | 1.65 |  | CH=H_4_PteGlu |  |
| CH_3_-H_4_PteGlu_4_ | 15.49 | 4.44E+08 | 2.22E+08 | 15.44 | 15.54 | 0.10 | 0.52 |  |  |  |
| CHO-H_4_PteGlu_4_ | 15.56 | 1.80E+08 | 8.99E+07 | 15.64 | 15.70 | 0.06 | 0.59 |  | CH_3_-H_4_PteGlu | -2.12 |
| CHO-H_4_PteGlu_5_ | 15.62 | 2.57E+08 | 1.29E+08 | 15.61 | 15.67 | 0.06 | 0.91 |  | CH_2_-H_4_PteGlu |  |
| CHO-H_4_PteGlu_6_ | 15.72 | 1.91E+08 | 9.54E+07 | 15.71 | 15.78 | 0.07 |  |  |  |  |
| CH=H_4_PteGlu | 15.68 | 5.08E+08 | 2.54E+08 | 15.63 | 15.73 | 0.10 |  |  |  |  |
| **HILIC method II.acidic** | | | | | | | | | | |
| CH_3_-H_4_PteGlu | 17.08 | 3.09E+08 | 1.54E+08 | 17.03 | 17.17 | 0.14 | 0.08 |  | PteGlu | 1.82 |
| PteGlu | 17.1 | 1.97E+08 | 9.87E+07 | 17.04 | 17.19 | 0.15 | 0.27 |  | H_2_PteGlu |  |
| CHO-H_4_PteGlu | 17.17 | 3.06E+08 | 1.53E+08 | 17.09 | 17.25 | 0.16 | 1.18 |  |  |  |
| CH_2_-H_4_PteGlu | 17.45 | 2.80E+08 | 1.40E+08 | 17.38 | 17.5 | 0.12 | 0.26 |  | H_2_PteGlu | 1.77 |
| H_2_PteGlu | 17.5 | 2.66E+08 | 1.33E+08 | 17.44 | 17.55 | 0.11 | 1.77 |  | H_4_PteGlu |  |
| H_4_PteGlu | 17.83 | 1.22E+08 | 6.12E+07 | 17.77 | 17.88 | 0.11 | 2.68 |  |  |  |
| CH_3_-H_4_PteGlu_2_ | 18.33 | 6.81E+08 | 3.40E+08 | 18.28 | 18.39 | 0.11 | 3.96 |  | CH_2_-H_4_PteGlu | -9.05 |
| CH_3_-H_4_PteGlu_3_ | 19.17 | 6.87E+08 | 3.44E+08 | 19.12 | 19.26 | 0.14 | 3.16 |  | CH=H_4_PteGlu |  |
| CH_3_-H_4_PteGlu_4_ | 19.84 | 5.35E+08 | 2.67E+08 | 19.79 | 19.90 | 0.11 | 1.13 |  |  |  |
| CHO-H_4_PteGlu_4_ | 20.09 | 1.88E+08 | 9.42E+07 | 20.05 | 20.20 | 0.15 | 1.14 |  | CH_3_-H_4_PteGlu | -1.68 |
| CHO-H_4_PteGlu_5_ | 20.38 | 2.69E+08 | 1.34E+08 | 20.33 | 20.48 | 0.15 | 1.77 |  | CH_2_-H_4_PteGlu |  |
| CHO-H_4_PteGlu_6_ | 20.83 | 2.52E+08 | 1.26E+08 | 20.79 | 20.94 | 0.15 |  |  |  |  |
| CH=H_4_PteGlu | 19.52 | 1.02E+09 | 5.08E+08 | 19.41 | 19.56 | 0.15 |  |  |  |  |
| **RPLC method I** | | | | | | | | | | |
| CH=H_4_PteGlu | 4.438 | 1.62E+08 | 8.12E+07 | 4.37 | 4.48 | 0.11 | 5.53 |  | PteGlu | -1.26 |
| H_4_PteGlu | 5.4221 | 1.77E+06 | 8.84E+05 | 5.4 | 5.5 | 0.10 | 3.07 |  | H_2_PteGlu |  |
| CH_3_-H_4_PteGlu | 6.0466 | 1.41E+08 | 7.06E+07 | 5.97 | 6.11 | 0.14 | 1.36 |  |  |  |
| CHO-H_4_PteGlu | 6.5289 | 1.32E+07 | 6.59E+06 | 6.37 | 6.65 | 0.28 | 0.45 |  | H_2_PteGlu | -7.39 |
| H_2_PteGlu | 6.6741 | 1.16E+07 | 5.80E+06 | 6.65 | 6.75 | 0.10 | N/A |  | H_4_PteGlu |  |
| pABGlu | N/A | N/A | N/A | N/A | N/A | N/A | N/A |  |  |  |
| CH_2_-H_4_PteGlu | 6.8866 | 6.46E+07 | 3.23E+07 | 6.85 | 7 | 0.15 | 0.14 |  | CH_2_-H_4_PteGlu | 11.11 |
| PteGlu | 6.92 | 3.42E+05 | 1.71E+05 | 6.87 | 7.00 | 0.13 | -1.63 |  | CH=H_4_PteGlu |  |
| CH_3_-H_4_PteGlu_2_ | 6.3532 | 4.63E+07 | 2.32E+07 | 6.28 | 6.56 | 0.28 | 1.87 |  |  |  |
| CH_3_-H_4_PteGlu_3_ | 7.751 | 8.54E+06 | 4.27E+06 | 7.6 | 8.20 | 0.60 | N/A |  | CH_3_-H_4_PteGlu | -3.42 |
| CH_3_-H_4_PteGlu_4_ | N/A | N/A | N/A | N/A | N/A | N/A | N/A |  | CH_2_-H_4_PteGlu |  |
| CHO-H_4_PteGlu_4_ | N/A | N/A | N/A | N/A | N/A | N/A | N/A |  |  |  |
| CHO-H_4_PteGlu_5_ | N/A | N/A | N/A | N/A | N/A | N/A | N/A |  |  |  |
| CHO-H_4_PteGlu_6_ | N/A | N/A | N/A | N/A | N/A | N/A | N/A |  |  |  |
| **RPLC method II** | | | | | | | | | | |
| CH=H_4_PteGlu | 2.6404 | 8.69E+08 | 4.34E+08 | 2.59 | 2.65 | 0.06 | 6.58 |  | PteGlu | -1.15 |
| H_4_PteGlu | 5.2045 | 2.97E+06 | 1.48E+06 | 5.06 | 5.46 | 0.40 | 2.59 |  | H_2_PteGlu |  |
| CH_3_-H_4_PteGlu | 6.9636 | 2.45E+07 | 1.23E+07 | 6.89 | 7.29 | 0.40 | 1.27 |  |  |  |
| CHO-H_4_PteGlu | 7.9092 | 1.60E+07 | 8.00E+06 | 7.67 | 8.15 | 0.48 | 0.31 |  | H_2_PteGlu | -4.54 |
| H_2_PteGlu | 8.1304 | 5.03E+07 | 2.51E+07 | 8.02 | 8.38 | 0.36 | 0.24 |  | H_4_PteGlu |  |
| pABGlu | 8.4027 | 3.45E+06 | 1.72E+06 | 7.95 | 8.95 | 1.00 | 0.31 |  |  |  |
| CH_2_-H_4_PteGlu | 8.734 | 7.29E+06 | 3.65E+06 | 8.67 | 8.93 | 0.26 | 0.24 |  | CH_2_-H_4_PteGlu | 22.47 |
| PteGlu | 8.87 | 4.24E+07 | 2.12E+07 | 8.80 | 9.20 | 0.40 | 0.10 |  | CH=H_4_PteGlu |  |
| CH_3_-H_4_PteGlu_2_ | 8.9497 | 6.26E+06 | 3.13E+06 | 8.82 | 9.40 | 0.58 | N/A |  |  |  |
| CH_3_-H_4_PteGlu_3_ | 15.77 | N/A | N/A | N/A | N/A | N/A | N/A |  | CH_3_-H_4_PteGlu | -3.17 |
| CH_3_-H_4_PteGlu_4_ | N/A | N/A | N/A | N/A | N/A | N/A | N/A |  | CH_2_-H_4_PteGlu |  |
| CHO-H_4_PteGlu_4_ | N/A | N/A | N/A | N/A | N/A | N/A | N/A |  |  |  |
| CHO-H_4_PteGlu_5_ | N/A | N/A | N/A | N/A | N/A | N/A | N/A |  |  |  |
| CHO-H_4_PteGlu_6_ | N/A | N/A | N/A | N/A | N/A | N/A | N/A |  |  |  |

# **Supplementary References**

1. Kwon YK, Lu W, Melamud E, Khanam N, Bognar A, Rabinowitz JD (2008) A domino effect in antifolate drug action in Escherichia coli. Nat Chem Biol 4:602–608. https://doi.org/10.1038/nchembio.108

2. Lu W, Kwon YK, Rabinowitz JD (2007) Isotope Ratio-Based Profiling of Microbial Folates. J Am Soc Mass Spectrom 18:898–909. https://doi.org/10.1016/j.jasms.2007.01.017

3. Schittmayer M, Birner-Gruenberger R, Zamboni N (2018) Quantification of Cellular Folate Species by LC-MS after Stabilization by Derivatization. Anal Chem 90:7349–7356. https://doi.org/10.1021/acs.analchem.8b00650

4. Osborn MJ, Talbert3 PT, Huennekens FM (1960) The Structure of “Active Formaldehyde” (N5,N10-Methylene Tetrahydrofolic Acid). J Am Chem Soc 82:4921–4927

5. Jagerstad M, Jastrebova J (2014) 5,10-Methylene-tetrahydrofolate dissociates into tetrahydrofolate and formaldehyde at physiological pH and acidic pH, typical conditions used during sample extraction and LC-MS/MS analysis of biological samples. Biomedical Chromatography 28:1041–1042. https://doi.org/10.1002/bmc.3214

6. Baggott JE (2000) Hydrolysis of 5,10-methenyltetrahydrofolate to 5-formyltetrahydrofolate at pH 2.5 to 4.5. Biochemistry 39:14647–14653. https://doi.org/10.1021/bi001362m

7. Chen L, Ducker GS, Lu W, Teng X, Rabinowitz JD (2017) An LC-MS chemical derivatization method for the measurement of five different one-carbon states of cellular tetrahydrofolate. Anal Bioanal Chem 409:5955–5964. https://doi.org/10.1007/s00216-017-0514-4

8. Van Haandel L, Becker M, Williams T, Stobaugh J, Leeder JS (2012) Comprehensive quantitative measurement of folate polyglutamates in human erythrocytes by ion pairing ultra-performance liquid chromatography/tandem mass spectrometry. Rapid Communications in Mass Spectrometry 26:1617–1630. https://doi.org/10.1002/rcm.6268

9. Garratt LC, Ortori CA, Tucker GA, Sablitzky F, Bennett MJ, Barrett DA (2005) Comprehensive metabolic profiling of mono- and polyglutamated folates and their precursors in plant and animal tissue using liquid chromatography/negative ion electrospray ionisation tandem mass spectrometry. Rapid Communications in Mass Spectrometry 19:2390–2398. https://doi.org/10.1002/rcm.2074

10. Gmelch L, Wirtz D, Witting M, Weber N, Striegel L, Schmitt-Kopplin P, Rychlik M (2020) Comprehensive vitamer profiling of folate monoand polyglutamates in baker’s yeast (Saccharomyces cerevisiae) as a function of different sample preparation procedures. Metabolites 10:1–19. https://doi.org/10.3390/metabo10080301

11. Schober AF, Mathis AD, Ingle C, Park JO, Chen L, Rabinowitz JD, Junier I, Rivoire O, Reynolds KA (2019) A Two-Enzyme Adaptive Unit within Bacterial Folate Metabolism. Cell Rep 27:3359-3370.e7. https://doi.org/10.1016/j.celrep.2019.05.030

12. Luo S, Duan H, Zou Y, Qiu R, Wang C (2017) Quantification of Total Folate, Folate Species and Polyglutamyl Folate Distribution in Winged Beans (Psophocarus tetragonolobus (L) DC) from Different Cultivars and Growth Stages by Ultra-High Performance Liquid Chromatography Tandem Mass Spectrometry

13. Kopp M, Dürr K, Steigleder M, Clavel T, Rychlik M (2016) Development of stable isotope dilution assays for the quantitation of intra- and extracellular folate patterns of Bifidobacterium adolescentis. J Chromatogr A 1469:48–59. https://doi.org/10.1016/j.chroma.2016.09.048

14. Wang C, Riedl KM, Schwartz SJ (2010) A liquid chromatography-tandem mass spectrometric method for quantitative determination of native 5-methyltetrahydrofolate and its polyglutamyl derivatives in raw vegetables. J Chromatogr B Analyt Technol Biomed Life Sci 878:2949–2958. https://doi.org/10.1016/j.jchromb.2010.08.043

15. Leung KY, De Castro SCP, Cabreiro F, Gustavsson P, Copp AJ, Greene NDE (2013) Folate metabolite profiling of different cell types and embryos suggests variation in folate one-carbon metabolism, including developmental changes in human embryonic brain. Mol Cell Biochem 378:229–236. https://doi.org/10.1007/s11010-013-1613-y

16. Ghergurovich JM, Xu X, Wang JZ, Yang L, Ryseck RP, Wang L, Rabinowitz JD (2021) Methionine synthase supports tumour tetrahydrofolate pools. Nat Metab 3:1512–1520. https://doi.org/10.1038/s42255-021-00465-w

17. Annibal A, Tam H, Latza C, Antebi A (2019) Comparison of ESI-MS/MS and APCI-MS methods for the quantification of folic acid analogs in C. elegans. Journal of Mass Spectrometry 54:316–327. https://doi.org/10.1002/jms.4337

18. Zhang H, Jha AB, Warkentin TD, Vandenberg A, Purves RW (2018) Folate stability and method optimization for folate extraction from seeds of pulse crops using LC-SRM MS. Journal of Food Composition and Analysis 71:44–55. https://doi.org/10.1016/j.jfca.2018.04.008

19. Nandania J, Kokkonen M, Euro L, Velagapudi V (2018) Simultaneous measurement of folate cycle intermediates in different biological matrices using liquid chromatography–tandem mass spectrometry. J Chromatogr B Analyt Technol Biomed Life Sci 1092:168–178. https://doi.org/10.1016/j.jchromb.2018.06.008

20. Zheng XH, Jiang LY, Zhao LT, Zhang QY, Ding L (2015) Simultaneous quantitation of folic acid and 5-methyltetrahydrofolic acid in human plasma by HPLC-MS/MS and its application to a pharmacokinetic study. J Pharm Anal 5:269–275. https://doi.org/10.1016/j.jpha.2015.05.004

21. Freisleben A, Schieberle P, Rychlik M (2003) Specific and sensitive quantification of folate vitamers in foods by stable isotope dilution assays using high-performance liquid chromatography-tandem mass spectrometry. Anal Bioanal Chem 376:149–156. https://doi.org/10.1007/s00216-003-1844-y
